# Genetically engineered two-warhead evasins provide a method to achieve precision targeting of disease-relevant chemokine subsets

**DOI:** 10.1038/s41598-018-24568-9

**Published:** 2018-04-20

**Authors:** Yara Alenazi, Kamayani Singh, Graham Davies, James R. O. Eaton, Philip Elders, Akane Kawamura, Shoumo Bhattacharya

**Affiliations:** 1 RDM Division of Cardiovascular Medicine, University of Oxford, Oxford, United Kingdom; 2 Department of Chemistry, University of Oxford, Oxford, United Kingdom

## Abstract

Both CC and CXC-class chemokines drive inflammatory disease. Tick salivary chemokine-binding proteins (CKBPs), or evasins, specifically bind subsets of CC- or CXC-chemokines, and could precisely target disease-relevant chemokines. Here we have used yeast surface display to identify two tick evasins: a CC-CKBP, P1243 from *Amblyomma americanum* and a CXC-CKBP, P1156 from *Ixodes ricinus*. P1243 binds 11 CC-chemokines with K_d_ < 10 nM, and 10 CC-chemokines with K_d_ between 10 and 100 nM. P1156 binds two ELR + CXC-chemokines with K_d_ < 10 nM, and four ELR + CXC-chemokines with K_d_ between 10 and 100 nM. Both CKBPs neutralize chemokine activity with IC_50_ < 10 nM in cell migration assays. As both CC- and CXC-CKBP activities are desirable in a single agent, we have engineered “two-warhead” CKBPs to create single agents that bind and neutralize subsets of CC and CXC chemokines. These results show that tick evasins can be linked to create non-natural proteins that target subsets of CC and CXC chemokines. We suggest that “two-warhead” evasins, designed by matching the activities of parental evasins to CC and CXC chemokines expressed in disease, would achieve precision targeting of inflammatory disease-relevant chemokines by a single agent.

## Introduction

The 47 structurally related secreted proteins that form the chemokine family differ in the spacing of cysteine residues at the N-terminus, and based on this, are classified as CC, CXC, CX3C or XC^1^. Of these, the 27 CC and 17 CXC chemokines^1^ form the majority. Multiple CC and CXC chemokines are typically expressed in tissues affected in inflammatory disease^2^. The combinatorial effects of these chemokines are responsible for the recruitment of innate and adaptive immune cells including monocytes, neutrophils, eosinophils, and lymphocytes to sites of inflammation^3–5^. The synergistic effects between certain CC and CXC chemokines are well documented, for instance, in monocyte, lymphocyte and neutrophil migration (reviewed in^6^), and the functional importance of certain CC-CXC chemokine interactions has recently been demonstrated^7^. The chemokine network has been difficult to therapeutically target using monovalent agents such as monoclonal antibodies or small molecules^8,9^. Mechanisms underlying the robustness of the network include expression of multiple chemokines in tissues affected by inflammatory disease, the ability of chemokines to interact with several chemokine receptors, expression of multiple receptor types on leukocytes and immune cells^10^, synergy and interactions between different chemokines^6,7^, and feed-forward loops where inflammatory cells recruited to diseased tissue secrete more chemokines, thereby amplifying the inflammatory response^11^.

A number of pathogens and parasitic organisms, including viruses, helminths and ticks, produce structurally unrelated chemokine binding proteins (CKBPs) that target multiple chemokines (reviewed in^9,12^). Viral and helminth CKBPs studied to date, with the exceptions of R17^13^ and 35-kDa/vCCI^14^, typically do not discriminate between CC and CXC chemokines^12,15–17^. Tick CKBPs or evasins, originally identified by Proudfoot *et al*. from *Rhipicephalus sanguineus*^18^, fall into two structurally unrelated classes that do discriminate between CC and CXC chemokines, and moreover, are also selective in their binding within either chemokine class. Evasins 1 and 4 solely bind distinctive subsets of CC chemokines, whereas evasin 3 binds only a subset of CXC chemokines. The convergent evolution of structurally unrelated peptides in diverse organisms that target the multiple nodes in the chemokine network, provides strong evidence that such one-to-many targeting is an effective strategy to disrupt chemokine-driven inflammation. Supporting this idea, the potential therapeutic efficacy of viral^15^, helminth^16^ and tick^9^ CKBPs in inflammatory disease provides proof-of-concept of targeting of the chemokine network as a therapeutic approach.

The inadvertent targeting of chemokines that are not involved in the disease process by CKBPs could however increase the likelihood of off-target effects. The preferential binding of tick evasins to limited subsets of CC or CXC chemokines could provide a method to precisely target multiple disease-relevant chemokines without unnecessarily affecting other chemokines. To achieve this there is a great need to identify further tick CKBPs that bind different subsets of CC and CXC chemokines. Putative evasins have been identified in a variety of tick species using bioinformatic analyses of salivary transcriptome data^19–21^. To rapidly identify tick CKBPs that bind chemokines with high-affinity, we screened yeast surface display libraries, constructed to express putative tick evasins, with fluorescently labelled chemokines, and cloned 10 further evasins from *Rhipicephalus* and *Amblyomma* genera^22^. We also showed that these new evasins bound with high affinity to subsets of CC chemokines that range in number from 8–20 chemokines, and not to CXC chemokines. Similar to evasins 1 and 4, these new evasins share eight conserved cysteine residues. A common unifying feature of the these 10 evasins was the ability to bind CCL2 and CCL13, properties which were not observed in evasins 1 and 4^22^. Further novel evasins that bind CC chemokines were identified more recently from *Ixodes, Rhipicephalus* and *Amblyomma* genera by Hayward *et al*.^23^.

Here we describe the functional properties of two evasins identified by yeast surface display, P1243 from *Amblyomma americanum* (also described in Hayward *et al*.^23^ as AAM-02), which is a CC-chemokine binding CKBP homologous to previously described CC-CKBPs, and P1156 from *Ixodes ricinus*, a novel CXC-binding CKBP that is homologous to evasin 3. We show that P1243 has a distinctive CC-chemokine-binding specificity, binding a large number of CC-chemokines including CCL13, but not binding CCL2. P1156 binds CXCL1 and CXCL8 strongly, but in addition binds CXCL2, 3, 5 and 6. The availability of different evasins that target subsets of CC and CXC chemokines in a one-to-many fashion provides the opportunity to use them in combination to treat inflammatory disease characterized by CC and CXC chemokine expression. The development of such therapeutics is however complicated by performance issues including risks and benefits of each individual agent in a combination^24^. These considerations prompted us to explore the possibility that the two distinct classes of tick evasins could be fused into a single agent. We show here, for the first time, that tick CC- and CXC- CKBPs can be fused together into a single entity we term a “two-warhead” evasin that binds subsets of both CC and CXC chemokines.

## Results

### Screening of a yeast surface display library

**Figure 1:**
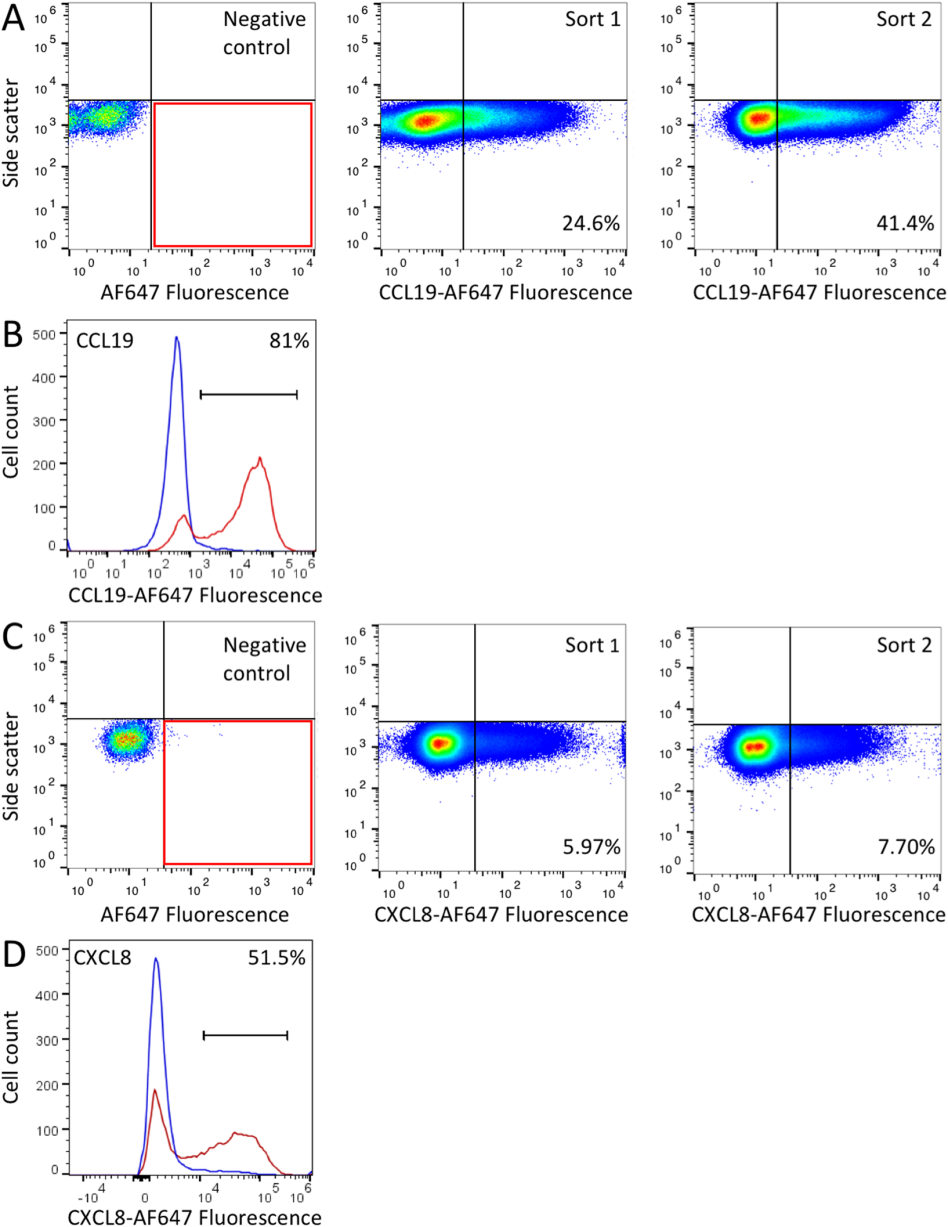
Yeast surface display screens. (**A**) Fluorescence profiles of tick putative CKBP yeast surface display library incubated with streptavidin-AF647 (negative control, left panel)) and with biotinylated CCL19 plus streptavidin-AF647, middle panel. The sorting gate (red box) was defined based on the negative control and was used to sort CKBP expressing yeast that bound biotinylated CCL19. The positive cells identified in the first sort were re-grown as a pool, and sorted once again to further enrich for chemokine binding yeast (right panel), and plated at low density to recover single clones. The y-axis shows side scatter and x-axis the fluorescence intensity on a log-scale. The proportions of cells within the sorting gate are indicated as a percentage. (**B**) Representative fluorescence profile (red) confirming the binding of a P1243 expressing yeast clone isolated in the above screen to biotinylated CCL19 and streptavidin-AF647. The fluorescence profile of control yeast with vector expressing the surface display tag alone are shown in blue. Y-axis shows cell count, and x-axis shows the fluorescence intensity on a log-scale. Positive cells with fluorescence exceeding that of the control are indicated as a percentage. The bar indicates the gate used. (**C**) Fluorescence profiles as above in (**A**), but using biotinylated CXCL8 plus streptavidin-AF647 as probe. (**D**) Representative fluorescence profile (red) confirming the binding of a P1156 expressing yeast clone isolated in the above screen to biotinylated CXCL8 and streptavidin-AF647. The fluorescence profile of control yeast with vector expressing the surface display tag alone are shown in blue. Y-axis shows cell count, and x-axis shows the fluorescence intensity on a log-scale. Positive cells with fluorescence exceeding that of the control are indicated as a percentage. The bar indicates the gate used.

We screened a yeast surface display library expressing 352 putative tick CKBPs^22^ using fluorescence activated cell sorting (FACS) with biotinylated CC and CXC chemokines followed by streptavidin–AlexaFluor (AF)647 to label and sort yeast cells that had bound the chemokine. The 352 putative CKBPs were cloned as a pool into yeast surface display vectors, with either N-terminus (AGA2) or C-terminus (SAG1 or AGA2) surface display tags. The pooled library was transformed into yeast, and cells were labelled with biotinylated chemokine and streptavidin-AF647. Labelled cells were sorted by FACS. We used a gate determined by omitting the chemokine to identify and recover a population of positive cells (Fig. 1A,C). Positive cells recovered in the first round were plated and re-grown, and sorted once again as above to further enrich for true positive clones. The sorted cells were plated at low density to enable picking of individual yeast clones. As part of our established screening protocol, the individual yeast clones were next re-tested by labelling with the biotinylated chemokine used in the screen and streptavidin – Alexa 647, and compared to negative control yeast that only expressed the relevant surface display tag (Fig. 1B,D). Consistent with observations from our previous studies^22^, and other yeast display methods^25^, we noted that a proportion of yeast do not display the protein. Library plasmids were recovered from clones that successfully re-tested positive, and were sequenced to identify the peptide. Through the screening process we identified two novel evasins, P1243 and P1156. P1243 was identified in screens that used CCL19 (Fig. 1A,B), and also CCL17, CCL4, CCL15, and CCL18 (data not shown). P1156 was identified in a screen performed using CXCL8 (Fig. 1C,D).

### Sequence analysis of P1243

**Figure 2:**
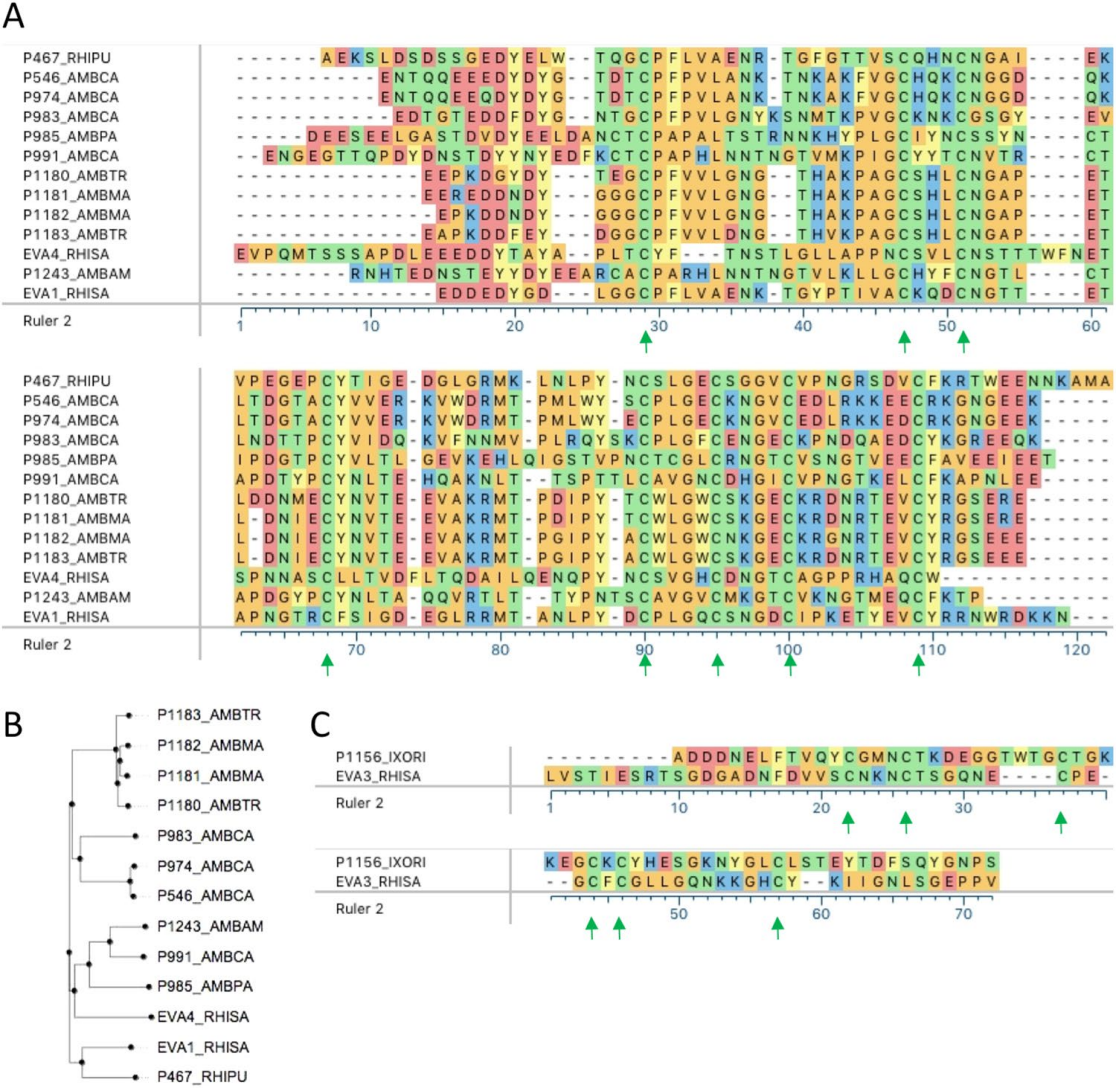
Analysis of P1243 and P1156. (**A**) CLUSTAL-W alignment of P1243 together with published CC-chemokine binding evasins. Protein sequence prefix indicates the identity, and suffix indicate the tick species as follows: AMBPA, AMBMA, AMBTR, AMBCA, - *Amblyomma parvum, maculatum, triste* and *cajennense* respectively, and RHIPU and RHISA – *Rhipicephalus pulchellus* and *sanguineus* respectively. Physicochemical properties are used to color code amino acid residues. Green arrows indicate the eight conserved Cys residues that form disulfide bonds in EVA1_RHISA (evasin 1). (**B**) Sequence similarity based phylogenetic tree of the “C8” CKBPs. (**C**) CLUSTAL-W alignment of P1156 together with EVA3_RHISA. Protein sequence prefix indicates the identity, and suffix indicate the tick species as follows: IXORI – *Ixodes ricinus*, and RHISA – *Rhipicephalus sanguineus*. Physicochemical properties are used to color code amino acid residues. The green arrows indicate the six Cys residues that are conserved in EVA3_RHISA (evasin 3).

P1243 has 98 amino acid residues with a predicted molecular weight of 10.9 kDa, and predicted pI of 6.94. Glycosylation site prediction indicates seven N-glycosylation sites. Alignment of P1243 with that of other published CC-chemokine binding tick CKBPs shows that it retains the eight Cys residues predicted to form disulfide bonds in evasin 1, and their arrangement is consistent with motif C-x(14,17)-C-x(3)-C-x(11,16)-C-x(17,20)-C-x(4)-C-x(4)-C-x(8)-C that we have previously described^22^ (Fig. 2A). Sequence-based phylogeny reveals that P1243 is most closely related to P991 (Fig. 2B), with which it shares 67% identity over an alignment length of 93 residues and P985 with which it shares 48% identity over 89 residues. It shares only 33% identity with evasin 1, over an alignment length of 62 residues. There is no significant homology to evasin 3. The Pro residue in evasin 1 that immediately follows the first Cys and targets the disulfide bond in CCL3^13^ is conserved in P1243. The conservation of the Cys residues in P1243 with evasin 1 suggested that they could potentially form a similar backbone disulfide bonded architecture. We explored this using homology modelling using the structure of evasin 1 in complex with the chemokine CCL3^26^. The Cys disulfide bond architecture observed in evasin 1 is conserved in P1243 (Supplementary Material Fig. S1).

### Sequence analysis of P1156

P1156 has 80 amino acid residues, with a predicted molecular weight of 8.8 kDa, and predicted pI of 4.7. It has two predicted N (at positions 16 and 61) and two predicted O-glycosylation sites at positions 63 and 75. It has 30% identity with evasin 3 over an alignment length of 43 residues, but no significant homology to evasins 1 or 4. An alignment of P1156 with EVA3_RHISA (evasin 3) is shown in Fig. 2C, and shows that it has six Cys residues like evasin 3.

### Expression of P1243 and P1156

**Figure 3:**
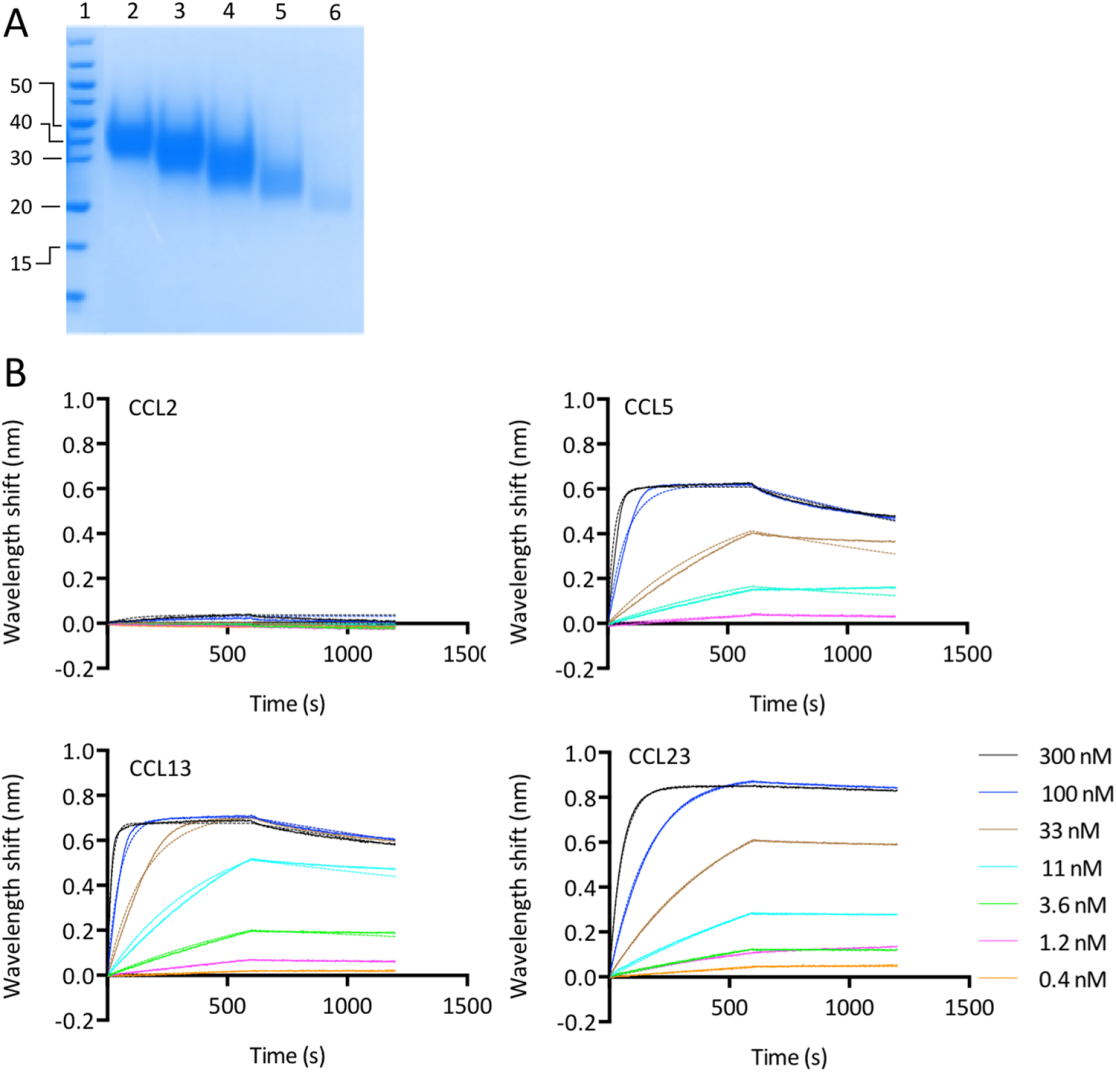
Characterization of P1243. (**A**) Colloidal Coomassie stained SDS-polyacrylamide gel showing size exclusion column fractions (lanes 2–6) obtained following nickel affinity chromatography for P1243. Molecular weight ladder (kDa) is shown in lane 1. The gel image was cropped for clarity and the uncropped image is provided in Fig.S5. (**B**) Biolayer interferometry sensorgrams of P1243 binding to indicated chemokines. Plots display wavelength shift (y-axis, nm) versus time (x-axis, seconds). Solid lines indicate collected data, dashed lines indicate fitted data.

**Figure 4:**
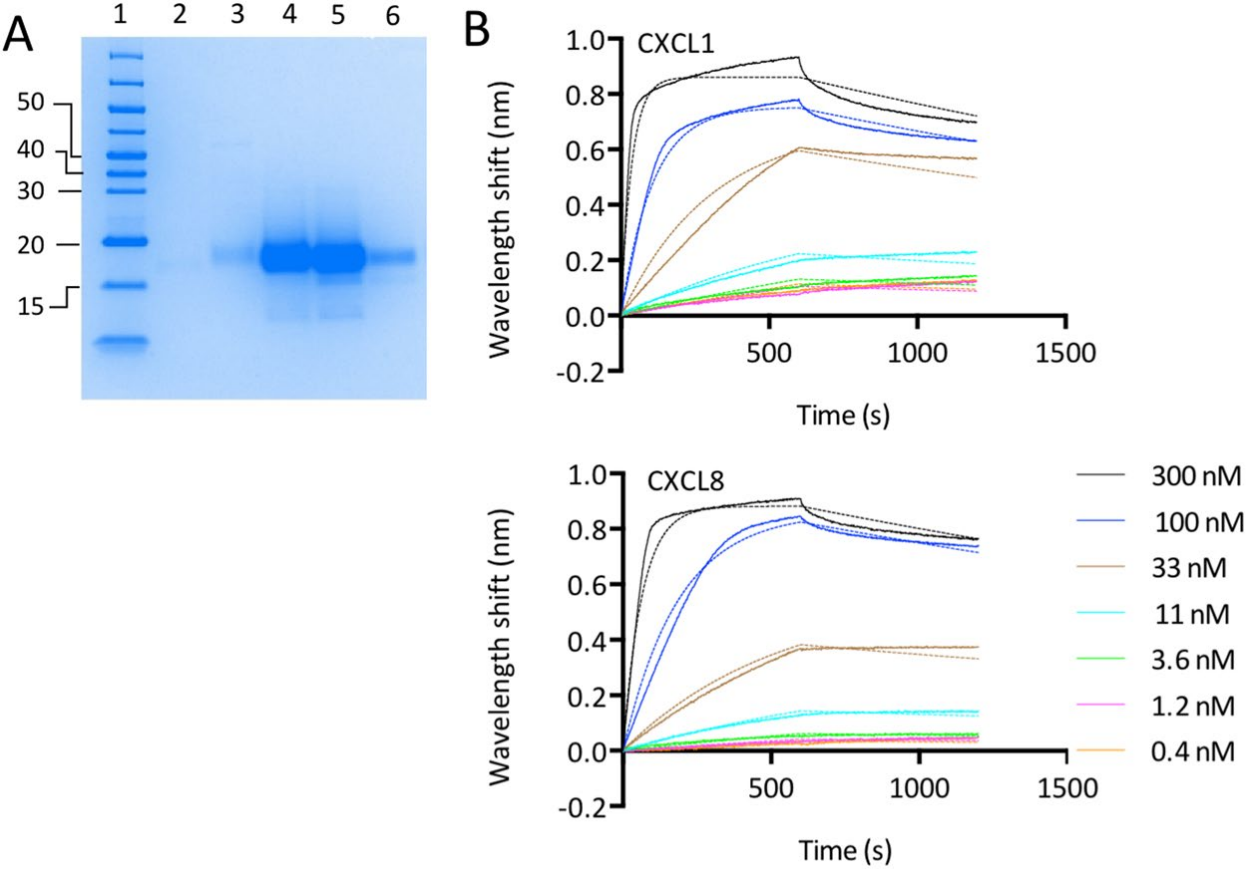
Characterization of P1156. (**A**) Colloidal Coomassie stained SDS-polyacrylamide gel showing size exclusion column fractions (lanes 3–6) obtained following nickel affinity chromatography for P1156. Molecular weight ladder (kDa) is shown in lane 1. The gel image was cropped for clarity and the uncropped image is provided in Fig.S5. (**B**) Biolayer interferometry sensorgrams of P1156 binding to indicated chemokines. Plots display wavelength shift (y-axis, nm) versus time (x-axis, seconds). Solid lines indicate collected data, dashed lines indicate fitted data.

P1243 was expressed as a secreted protein from mammalian cells essentially as described previously^22^, and purified using a C-terminal StrepII:His purification tag by Ni-NTA affinity followed by size exclusion chromatography. P1243 protein in the size exclusion fractions migrated in a range of molecular weights ranging from 20–50 kDa (Fig. 3A). This is significantly greater than the predicted molecular weight of 10.9 kDa, and is consistent with the predicted glycosylation at multiple sites, and also with the range of molecular weights observed for other evasins expressed in mammalian cells reported by others and us previously^22,27^. The size exclusion fractions containing protein were pooled for subsequent assays. P1156 was expressed and purified (Fig. 4A), as described above for P1243. P1156 protein in the size exclusion column fractions migrated at ~17 kDa, significantly larger than its predicted molecular weight of 8.8 kDa, and again consistent with its predicted glycosylation. Fractions 4 and 5 were pooled and used for further assays.

### Characterization of P1243

**Figure 5:**
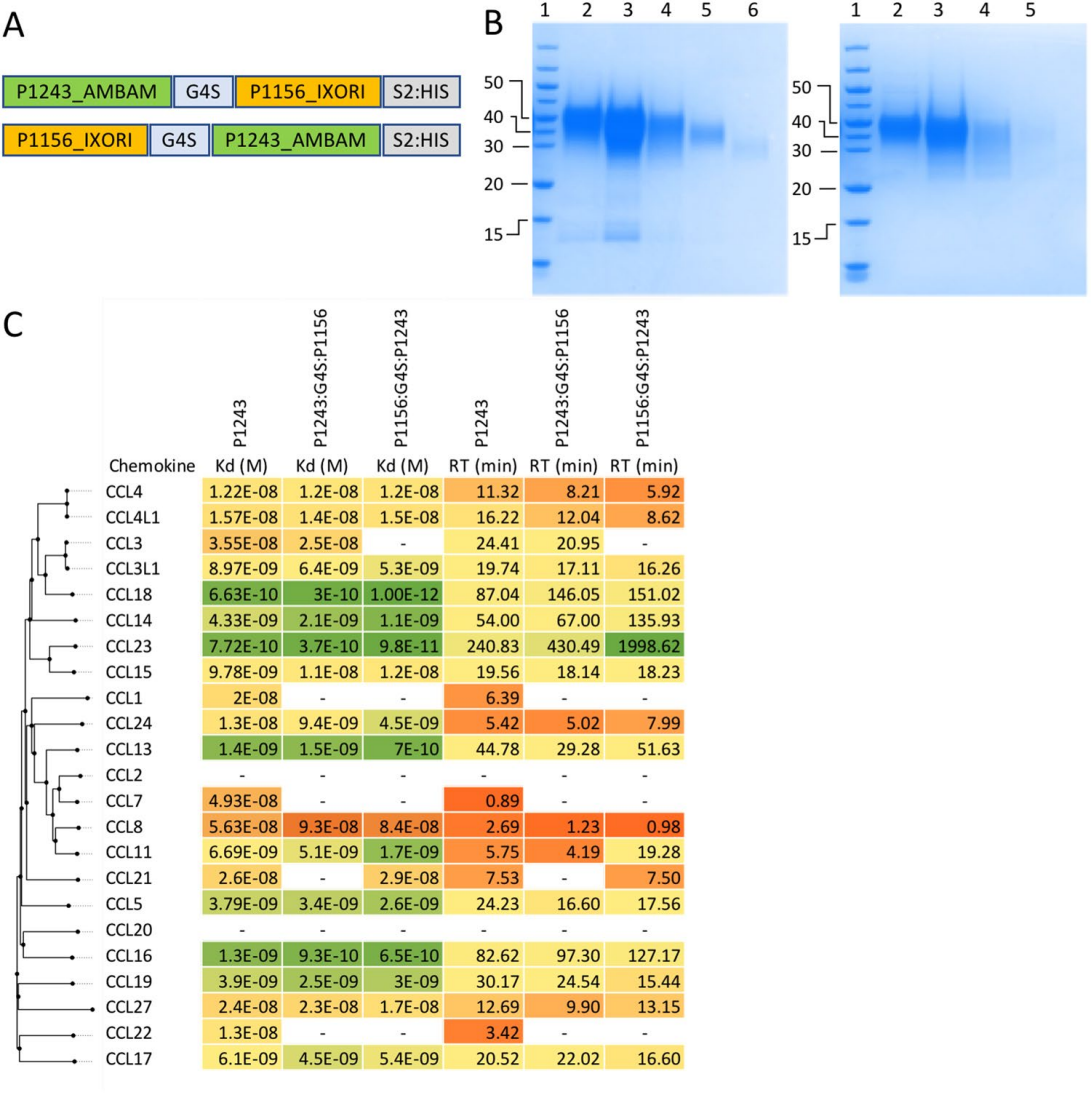
Characterization of single and “two-warhead” evasins. (**A**) Arrangement of the 2-warhead expression constructs P1243:G4S:P1156 and P1156:G4S:P1243 (not to scale). P1243 was engineered in-frame with a GGGGS (G4S) flexible linker to P1156. The construct was tagged at the C-terminus with a StrepII:8xHis purification tag (S2:HIS). (**B**) Colloidal Coomassie stained SDS-polyacrylamide gels showing size exclusion column fractions of P1243:G4S:P1156 (left) and P1156:G4S:P1243 (right) obtained following nickel affinity chromatography. Molecular weight ladder (kDa) is indicated in lane 1. The gel images were cropped for clarity and the uncropped images are provided in Fig.S5. (**C**) Binding affinities (K_d_, M) and target residence times (RT, minutes) of P1243, P1243:G4S:P1156 and P1156:G4S:P1243 to human CC-chemokines using biolayer interferometry. High affinity binding is indicated as shades of green, medium affinity as yellow, and low affinity as shades of orange. Chemokines are arranged by sequence-similarity based phylogeny. A dash (-) indicates that binding was either not detected (CCL2) or that K_d_ could not be determined. Affinity and target residence time in each case was calculated on data obtained at five or more chemokine concentrations.

We next assayed the binding of P1243 to a panel of chemokines using biolayer interferometry^28^. P1243 was immobilized on the sensor through the C-terminal His tag. An initial cross-binding screening assay performed at 300 nM chemokine concentration indicated that P1243 bound a number of CC chemokines, certain CXC chemokines, and also CX3CL1 and XCL1 (Supplementary Material Fig. S2, Table S2). Binding affinities (K_d_) for P1243 were next determined using biolayer interferometry titration studies to chemokines identified as potentially binding P1243 in the cross-binding screen. In the titration assay, for several chemokines, binding was only detected at 300 nM and not at lower concentrations (data not shown). These were XCL1, CX3CL1, CXCL4, CXCL5 CXCL7, CXCL9, CXCL12 and CXCL14, and as curve fitting could not be performed, and we interpret these as low affinity binding and did not investigate them further. Higher affinity binding was identified for 21 CC chemokines where curve fitting could be performed. Example sensorgrams are shown in Fig. 3B, with summary K_d_ and target residence time data shown Fig. 5C. Notably, and in contrast to CC-binding evasins previously identified^22,26,29,30^, P1243 bound CCL13, but did not bind CCL2. We also noted that there was a remarkable variation in target residence time (calculated from the off rate) ranging from under one minute for CCL7, to over 240 minutes for CCL23.

### Characterization of P1156

**Figure 6:**
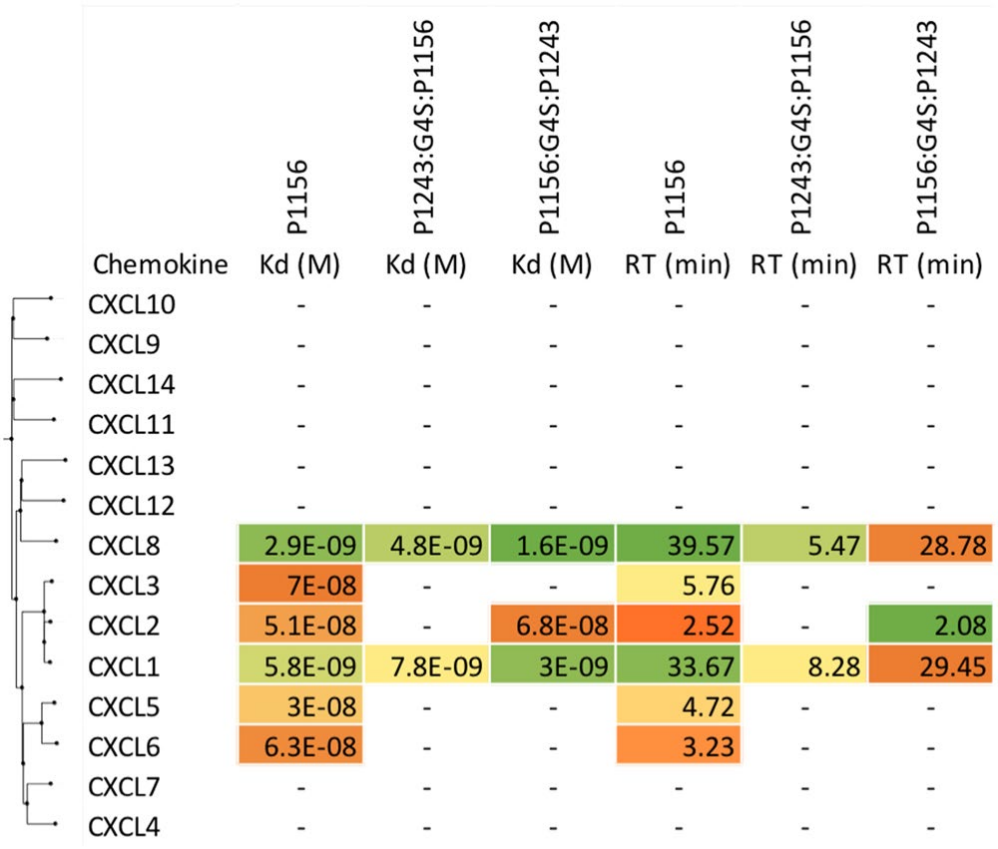
Characterization of CXC chemokine binding by single and “two-warhead” evasins. Binding affinities (K_d_, M) and target residence times (RT, minutes) of P1156, P1243:G4S:P1156 and P1156:G4S:P1243 to human CXC-chemokines using biolayer interferometry. High affinity binding is indicated as shades of green, medium affinity as yellow, and low affinity as shades of orange. Chemokines are arranged by sequence-similarity based phylogeny. A dash (-) indicates that either a titration study was not performed in this experiment (CXCL4, 7, 9, 10, 11, 12, 13, 14), or that binding could not be detected. Affinity and target residence time in each case was calculated on data obtained at five or more chemokine concentrations.

We next assayed the binding of P1156 to a panel of chemokines using biolayer interferometry as described above. An initial screening assay performed at 300 nM chemokine concentration indicated that P1156 bound only certain ELR + CXC chemokines and not CC, XC or CX3C chemokines (Supplementary Material Fig. S2). Binding affinities (K_d_) for P1156 were next determined using biolayer interferometry titration studies against CXC chemokines that were identified as potential binders in the cross-binding screen. P1156 bound a number of closely related human ELR + CXC chemokines, including CXCL1, 2, 3, 5, 6 and 8. Example sensorgrams are shown in Fig. 4B, with summary K_d_ and target residence time data shown in Fig. 6.

### Development of “two-warhead” evasins

We fused P1243 to P1156 via a flexible GGGGS linker in order to create the “two-warhead” evasins P1243:G4S:P1156 and P1156:G4S:P1243 (Fig. 5A). The length of the peptide in each case is 183 amino acid residues, with a predicted molecular weight of 20 kDa, and pI of 5.3. The “two-warhead” evasins were expressed and purified using a C-terminal His tag using Ni-NTA affinity purification as described previously for the individual evasins. To confirm that full length proteins were produced we performed N-terminal sequencing of the expressed proteins. This confirmed that the predicted first four residues matched the first four residues observed by N-terminal sequencing in each case (Supplementary Material Fig. S6). To confirm the integrity of the C-termini we used western blotting of glycosylated and deglycosylated two-warheads using anti-His antibody (Supplementary Material Fig. S7). This showed that glycosylated P1243:G4S:P1156 and deglycosylated P1156:G4S:P1243 could be detected readily. Surprisingly, deglycosylated P1243:G4S:P1156 and glycosylated P1156:G4S:P1243 could not be detected in these experiments. We do not have an explanation for this glycosylation-dependent variability except that such immunodetection issues have also been noted with other His-tagged recombinant proteins^31^. However, the purification on Ni-NTA affinity column, binding to the biolayer interferometry Ni-NTA sensor, together with the western blot data indicate that the C-terminal His-tag was intact. Together these experiments showed that the full-length two-warhead proteins were produced in each case. Analysis of the purified protein fractions showed that they migrated at higher molecular weights (between 30–60 kDa) than the expected molecular weight of 20 kDa (Fig. 5B). This is consistent with the predicted glycosylation for P1243 and for P1156. The purified protein fractions obtained for each “two-warhead” evasin were pooled for further analysis.

### Binding of “two-warhead” evasins to CC and CXC chemokines

We next assayed the binding of the “two-warhead” evasins to a panel of CC and CXC chemokines using biolayer interferometry, initially by determining if they bound at 100 nM chemokine concentration in a cross-binding screen (Supplementary Material Fig. S2). This suggested binding to several CC and CXC chemokines. In these studies, we found that P1243:G4S:P1156 appeared to bind certain chemokines (CXCL4, 7, 12, 13, 14) that were not bound by the parental evasins. In titration experiments we however found that this binding could not be confirmed (Supplementary Material Fig. S8), and did not follow this up further. No appreciable binding could be observed for XC or CX3C chemokines for either two-warhead evasin. We next performed titration assays in parallel to compare the binding of the parental evasins to the “two-warhead” evasins. The results showed that the “two-warhead” evasins bound several, but not all of the chemokines bound by the parental evasins (Figs 5C, 6). The chemokines most affected by the fusion were the ones that bound the parental evasin with lower affinity. For instance, the two-warhead evasins did not bind CCL1, CCL7, CCL22, CXCL3, CXCL5 or CXCL6, whereas the parental evasins clearly did. We also observed that the relative position of the two evasins in the construction affected the binding patterns to some extent. For example, P1156:G4S:P1243 bound CCL21 and CXCL2, but P1243:G4S:P1156 did not.

### Inhibition of CC chemokine function by individual and “two-warhead” evasins

**Figure 7:**
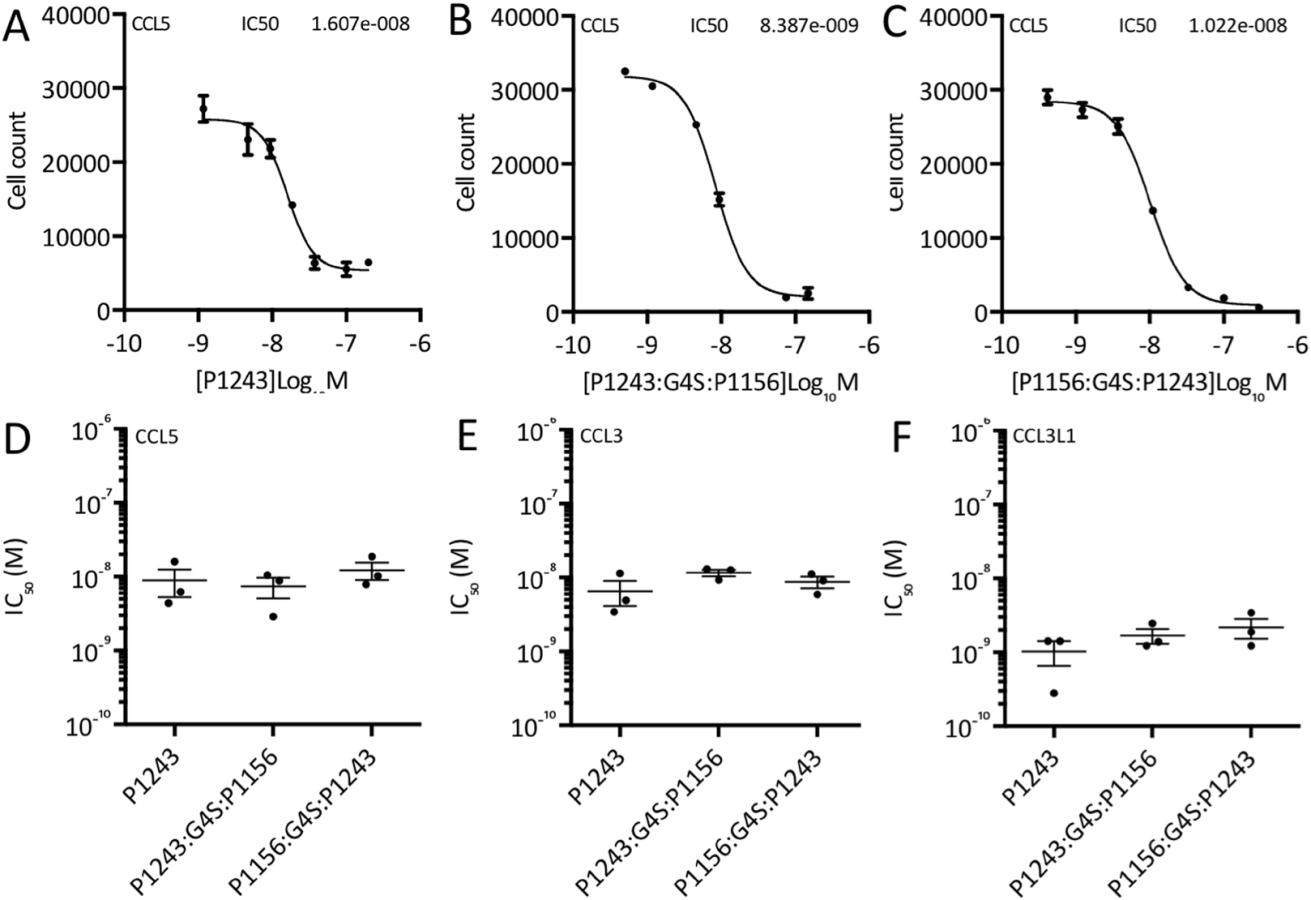
Functional inhibition of CC chemokines by individual and “two-warhead” evasins. (**A**–**C**) Neutralization of CCL5 induced THP-1 cell migration by P1243, P1243:G4S:P1156, and P1156:G4S:P1243 respectively. Y-axis shows cell count of THP-1 cells migrating through to the bottom chamber in response to EC_80_ dose of CCL5. In each case, data from a representative experiment are shown as mean ± s.e.m. of three technical replicates. X-axis shows CKBP concentration (Log_10_ Molar). IC_50_ values (M) indicated in each figure were estimated by fitting an agonist response curve with 4 parameters^22^. (**D**–**F**) Summary IC_50_ data (mean ± s.e.m., and individual data points from three biological replicates) of the indicated CKBPs against CCL5, CCL3 and CCL3L1 respectively. Y-axis shows IC_50_ (logarithmic scale, M), and x-axis shows each CKBP. CC chemokines were assayed using THP-1 cell migration. Each chemokine was assayed at its EC_80_ dose. There were no statistically significant differences between the mean IC_50_ values in each figure.

To explore if individual and “two-warhead” evasins would inhibit CC chemokine function, we monitored their effects on the migration of THP-1 human monocyte cells in response to human CC chemokines in 96 well Boyden chamber assays. We determined the effect of titrating in progressively increasing doses of evasins at the EC_80_ dose of each chemokine. Individual dose-response experiments against CCL5 are shown in Fig. 7A–C, from which IC_50_ data were calculated. Summary IC_50_ data for CCL3, CCL3L1 and CCL5, obtained from three biological replicates of such dose-response experiments are presented in Fig. 7D–F. Consistent with their ability to bind CCL3, CCL3L1, and CCL5, we found that the parental P1243 and “two-warhead” evasins also inhibited the function of these chemokines in a dose-dependent manner. Analysis of the summary data (Fig. 7D–F) indicated that the mean IC_50_ values for P1243, P1243:G4S:P1156, and P1156:G4S:P1243 respectively against CCL5 were 8.9E-9, 7.4E-9 and 1.2E-8 M, against CCL3 were 6.5E-9, 1.2E-8, and 8.8E-9 M, and against CCL3L1 were 1E-9, 1.7E-9, and 2.2E-9 M. There was no statistically significant difference in the ability of either “two-warhead” to inhibit these CC chemokines in comparison to the parental evasin P1243.

### Inhibition of CXC chemokine function by individual and “two-warhead” evasins

**Figure 8:**
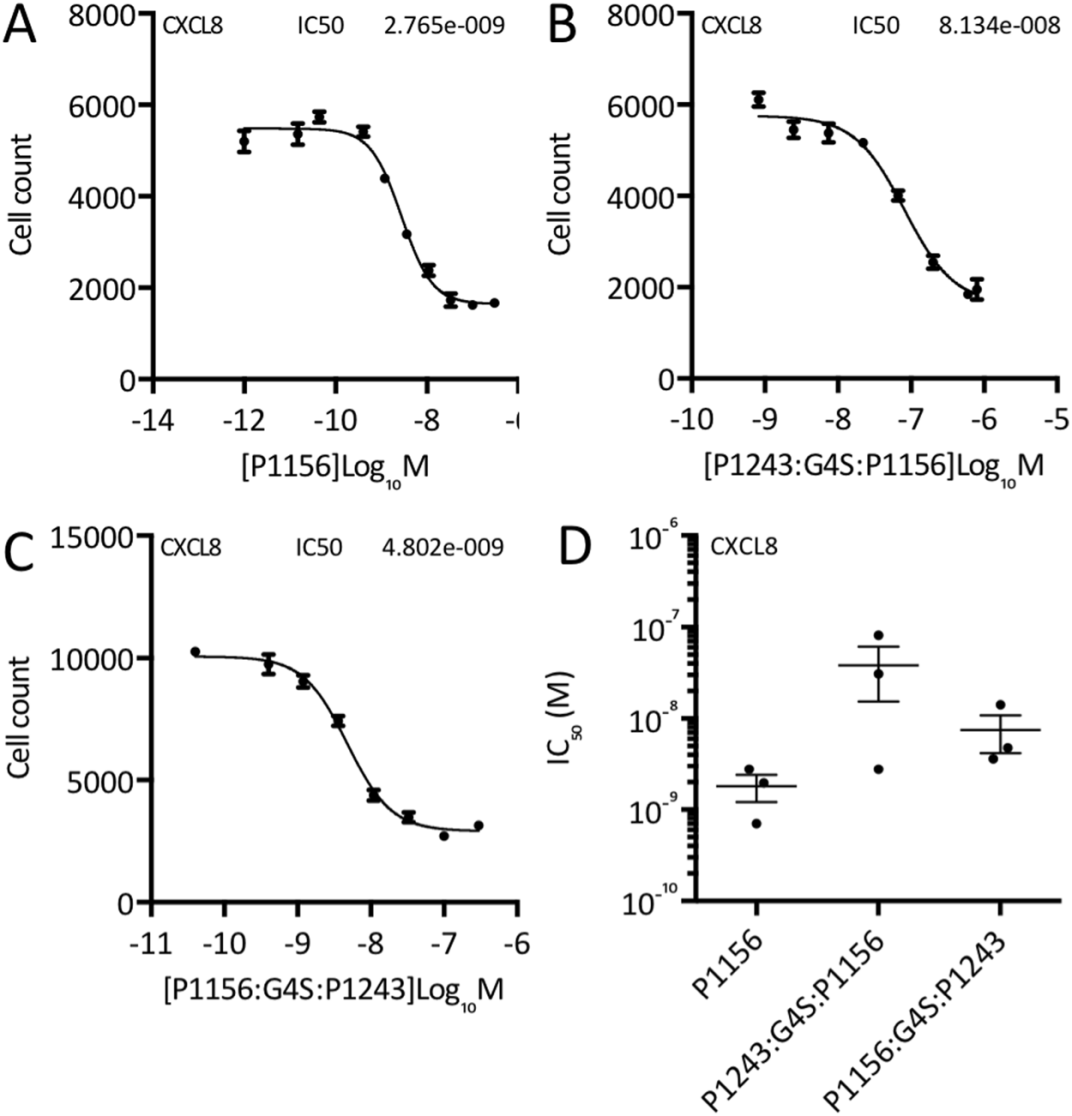
Functional inhibition of CXC chemokines by individual and “two-warhead” evasins. (**A**–**C**) Neutralization of CXCL8 induced granulocyte cell migration by P1156, P1243:G4S:P1156 and P1156:G4S:P1243 respectively. Y-axis shows cell count of granulocytes migrating through to the bottom chamber in response to EC_80_ dose of CXCL8. Data from a representative experiment are shown as mean ± s.e.m. of three technical replicates. X-axis shows CKBP concentration (Log_10_ Molar). IC_50_ values (M) indicated in each figure were estimated by fitting an agonist response curve with 4 parameters^22^. (**D**) Summary IC_50_ data (mean ± s.e.m., and individual data points from three biological replicates) of the indicated CKBPs against CXCL8. Y-axis shows IC_50_ (logarithmic scale, M), and x-axis shows each CKBP. CXCL8 was assayed using granulocyte cell migration at its EC_80_ dose. There were no statistically significant differences between the mean IC_50_ values.

We next examined the effect of the parental P1156 and “two-warhead” evasins on the migration of primary human granulocyte cells in response to CXCL8. Individual dose-response experiments are shown in Fig. 8A–C, from which IC_50_ data were calculated. Summary IC_50_ data obtained from three biological replicates of dose-response experiments are presented in Fig. 8D. Consistent with their ability to bind CXCL8, we found that the parental P1156 and “two-warhead” evasins also inhibited the function of CXCL8 in a dose-dependent manner. Analysis of the summary data (Fig. 8D) indicated that the mean IC_50_ values for P1156, P1243:G4S:P1156, and P1156:G4S:P1243 against CXCL8 were 1.8E-9, 3.8E-8 and 7.5E-9 M respectively. The differences between the evasins were not statistically significant.

## Discussion

Chemokine-driven inflammation plays a major role in pathogenesis of disease^2^. These include cardiovascular disorders such as myocardial infarction^32^, myocarditis^33^, atherosclerotic plaque^34^, and vasculitis^35^, neurological disorders such as stroke^36,37^, multiple sclerosis^38^, and Alzheimer disease^37^, gastrointestinal disorders including inflammatory bowel disease^37^, pancreatitis^39^, non-alcoholic steatohepatitis^40^ and other inflammatory hepatobiliary diseases^41–43^, lung diseases including idiopathic pulmonary fibrosis^44^, influenza^45,46^ and acute lung injury^47^, joint diseases such as rheumatoid arthritis^8^, and disorders of the skin including psoriasis^48^ and scleroderma^44^. Many of these disorders are characterized by an initiating insult such as ischemia in myocardial infarction and stroke, a viral infection in myocarditis and influenza, lipid deposition in the atherosclerotic plaque and non-alcoholic steatohepatitis, toxins such as paracetamol or alcohol in liver injury. The initiating insult results in the expression of chemokines and cytokines, and the ensuing inflammation can cause further additional damage to organs resulting in a vicious cycle of damage and further inflammation.

Typically, in these diseases, multiple CC and CXC class chemokines are expressed, and function synergistically to recruit different types of inflammatory cells. Examples of diseases where multiple CC and CXC chemokines are expressed include atherosclerosis^34^, rheumatoid arthritis^8^, and idiopathic pulmonary fibrosis^44^. The expression of multiple synergistically acting chemokines from different classes in diseased tissues is a factor that contributes to the difficulty in targeting chemokines using monovalent anti-chemokine therapeutics^8,9^. A wide range of pathogens and parasitic organisms ranging from viruses^15^, helminths^16^ and ticks^9^ produce structurally distinct chemokine-binding proteins (CKBPs) that target multiple chemokines. Pre-clinical trials using these CKBPs has provided proof-of-concept that targeting of multiple chemokines could be effective in inflammatory disease. In particular, tick CKBPs have been shown to be effective in pre-clinical models of arthritis, lung inflammation, colitis, pancreatitis, ischemia-reperfusion injury, plaque vulnerability, and psoriasis (reviewed in^9^).

Putative tick CKBPs have been identified in a variety of tick species using bioinformatic analyses of salivary transcriptome data^19–21^. In an effort to develop a therapeutic armamentarium of tick CKBPs that would provide the ability to target only disease-relevant chemokines without unnecessarily targeting irrelevant chemokines we have developed a yeast surface display approach to identify high-affinity interactors out of libraries of bioinformatically identified putative tick CKBPs^22^. Using this approach we identified 10 novel evasins, from *Rhipicephalus* and *Amblyomma* genera^22^. We also showed that these new evasins bound specifically to between eight (i.e. “narrow spectrum”) to 20 (i.e. “broad-spectrum”) CC chemokines, but not to CXC chemokines. A common unifying feature of the these 10 new evasins was their ability to bind CCL2 and CCL13, properties which were not observed in evasins 1 and 4^22^. These evasins contain eight cysteine residues that are conserved in evasins 1 and 4. Further novel eight cysteine or “C8” evasins that bind CC chemokines were identified more recently from *Ixodes, Rhipicephalus* and *Amblyomma* genera by Hayward *et al*.^23^.

In this study, we have identified and characterized a further C8 CC-CKBP, P1243. This was also recently reported and partially characterized by Hayward *et al*.^23^ as AAM-02, and shown to bind CCL11, CCL24, CCL26, CCL2, and CCL7. Our results indicate that P1243 binds 21 CC chemokines, of which 11 are bound with K_d_ < 10 nM, and so can be regarded as a “broad-spectrum” CC-chemokine inhibitor, akin to EVA4_RHISA^29^ and P991^22^. However, P1243, unlike P991, does not bind CCL2 at 300 nM, and we were unable to determine a K_d_ using our assay conditions (data not shown). This is consistent with data from Hayward *et al*.^23^ who show that the K_d_ for AAM-02 binding to CCL2 is 1000 nM. In comparison to P991, P1243 has a far shorter target residence time for chemokines that are bound in common, suggesting that a potential use may be to achieve short-term inhibition of the chemokine system, whereas P991 would achieve a longer-term inhibition. Analysis of CC-CKBPs show that they all retain the eight cysteine residues that form disulfide bonds in evasin 1^22^. Homology modelling of P1243 and other CC-CKBPs reported previously by us^22^ indicates that these Cys residues likely form disulfide bonds, and we thus refer to them as “C8” tick CKBPs. Like previously characterized CC-CKBPs, P1243 has multiple predicted glycosylation sites, and in keeping with this, migrates significantly slower than predicted by the calculated molecular weight of the peptide. As glycosylation does not appear to be required for chemokine recognition^26^, it may instead have other functions for instance reducing immunogenicity by creating a “glycan-shield”^49,50^, or improving protein stability^51^.

In this study, we have also identified a novel CXC-CKBP, P1156, which binds six ELR + CXC class chemokines, CXCL1, 2, 3, 5, 6 and 8, which have, in common, a Glu-Leu-Arg motif immediately preceding the first Cys residue, the ability to bind the receptors CXCR1 and/or CXCR2, and neutrophil trafficking activity^1^. P1156 is homologous to EVA3_RHISA^30^, and like it, binds CXCL1 and CXCL8 strongly. We have subsequently identified a number of additional evasins that have six Cys residues that align well with the six Cys residues of evasin 3 and bind CXC-chemokines. We therefore refer to these tick CKBPs collectively as “C6” CKBPs. It should be noted that the “C6” evasin P1156 described in this study is distinct from those described in Hayward *et al*.^23^. Like EVA3_RHISA, P1156 is also predicted to be glycosylated, and migrates significantly slower than predicted by the calculated molecular weight of the peptide.

The availability of several evasins that bind multiple CC and CXC chemokines provides an opportunity to match disease-relevant subsets of both CC and CXC chemokines, and develop novel therapeutics that would not target irrelevant chemokines. A disadvantage of tick CKBPs, however, is that their inability to target both CC and CXC chemokines would mean that combinations of “C8” CC- and “C6” CXC-CKBPs would need to be used. Such combinations of one or more agents require evaluation of the risks and benefits of each agent in the combination^24^, rendering the development of combination therapeutics more difficult. Inspired by the development of bispecific antibodies^52^, we explored the possibility that the two distinct classes of tick CKBPs could be fused into a single “two-warhead” agent. To achieve this, we linked P1243 with P1156 using a GGGGS linker to minimize steric hindrance and misfolding. The resultant “two-warhead” CKBPs retain most of the properties of the parental CKBPs, and can bind and neutralize subsets of both CC and CXC-chemokines. The mechanism of loss of some chemokine binding in the “two-warhead” CKBPs is unclear at present. One possible explanation is steric hindrance, and although this was not overcome by swapping the relative positions of P1243 and P1156, it could potentially be overcome by adopting longer linkers or other linker types^53^. A limitation of our current work is that we have only shown the effects of the two-warhead evasins in assays that measure chemokine effects on single cell types such as monocytes or granulocytes. In future work it would be important to develop *in vitro* assays that use different combinations of white blood cell types, for instance present in whole blood, and a mixture of chemokines, and also validate the two-warhead evasins usng *in vivo* assays of inflammation.

Applications of a “two-warhead” tick CKBP that binds both a CC chemokine and a CXC chemokine may include the therapeutic inhibition of chemokine signalling in any inflammatory disease associated with both CC and CXC chemokines. By examination of chemokine expression patterns in disease and the binding characteristics of the two-warhead CKBP P1243:G4S:P1156 reported in this paper, certain exemplar therapeutic indications would thus include myocarditis, myocardial infarction, atherosclerosis, vasculitis, idiopathic pulmonary fibrosis, inflammatory bowel disease and rheumatoid arthritis (Table S1). An alternative approach would be to combine a CC chemokine binding evasin with a CXCR1/CXCR2 antagonist such as SCH527123/diaminocyclobutandione-1^54^, which would block signalling by the neutrophil attracting chemokines blocked by P1156. However, as discussed previously, the development of such combination therapeutics is complicated by issues involving risks and benefits of each individual agent in a combination^24^.

In conclusion, we have shown that it is possible to target specific CC- and CXC-chemokine subsets by using a single agent that is created by a fusion of a tick CC- and a tick CXC-CKBP. As each tick CC- and CXC-CKBP has a remarkably distinctive chemokine binding profile, including narrow and broad-spectrum binding activities, the single agent created by fusing the two will also have a unique and distinctive CC and CXC-chemokine binding profile. This ability to tailor the chemokine binding profile of a “two-warhead” evasin by selecting parental evasins with appropriate CC and CXC binding activity, may provide a significant advantage over viral or helminth CKBPs where such ability to tailor binding properties has not yet been demonstrated. We suggest that novel “two-warhead” CKBPs, designed by matching the chemokine-subset binding properties of parental CKBPs to the CC and CXC chemokines expressed in a particular inflammatory disease, would achieve precision targeting of disease-relevant CC and CXC chemokine subsets by a single agent. We predict that such agents would be useful in the therapy of a broad range of inflammatory disease that are characterised by the combinatorial expression of CC and CXC chemokines.

## Experimental procedures

### Yeast surface display

Yeast surface display was performed exactly as described previously^22^. Briefly, EBY100 yeast transformed with tick transcriptome library plasmids were induced with galactose and labelled with biotinylated chemokines (Almac) and streptavidin-AF647, and then sorted using a MoFlo FACS system based on a gate defined by a negative control that omitted the chemokine. Cells obtained were regrown, and a second round of sorting was performed, following which cells were plated at low dilution to facilitate recovery of individual clones. Plasmid inserts from individual clones were amplified by PCR and sequenced to identify the insert. Individual yeast clones were also re-tested to confirm chemokine binding by FACS analyses using a 96-well ATTUNE NXT system (Life Technologies).

### Plasmids

P1243, P1243:G4S:P1156 and P1156:G4S:P1243 expression plasmids were constructed in plasmid pHLSec^55^ using PCR and infusion cloning as described^22^ and contained a C-terminal G4S linker, a strep II-8xHis protein purification tag (GGGGGSGGGGSGGASAWSHPQFEKLEHHHHHHHH^56^), and an N-terminal ETG sequence from pHLSec.The P1156 expression plasmid was constructed using idempotent parts using our laboratory’s adaptation of the GoldenGate/GoldenBraid cloning method^22^. Parts included the CAGGS promoter (from GenBank Sequence AB281497), Igk signal peptide^57^, StrepII:His tag (GGASAWSHPQFEKLEHHHHHHHH, pQE-Trisystem, Qiagen), and bovine growth hormone terminator (pcDNA3, Invitrogen). Following signal peptide cleavage the expected sequence at the N-terminus is DGG predicted by SignalP 3.0^58^.

### Protein sequence analysis

We used Clustal-W in Megalign Pro (DNAStar version 12.3.1, DNAStar Inc.), to construct CKBP sequence alignments and generate a sequence-similarity based phylogenetic tree which was exported to FigTree (version 1.4.2, http://tree.bio.ed.ac.uk/software/figtree/). Glycosylation sites were predicted using NetNGlyc1.0 (http://www.cbs.dtu.dk/services/NetNGlyc/) and NetOGlyc4.0 (http://www.cbs.dtu.dk/services/NetOGlyc/)^59^. Molecular weight and isoelectric point (pI) of proteins were calculated at ExPASy(http://web.expasy.org/compute_pi/). We used MUSCLE in Megalign Pro to construct chemokine sequence alignments and sequence-similarity based phylogenetic trees. Homology modelling was performed using MODELLER^60^, within PYMOD2.0^61^, using the evasin 1: CCL3 structure 3FPU^26^ as template. Disulfide bonds were identified using using the Protein Interaction Calculator^62^.

### Cell lines

HEK293F cells were a gift from Nicola Burgess-Brown (University of Oxford). THP-1 cells were purchased from Sigma. A MycoAlert™ (Lonza) kit was used to confirm freedom from mycoplasma contamination. Cells were functionally authenticated by protein production and chemokine – induced migration for HEK293F and THP-1 respectively.

### CKBP production

CKBPs were produced as described previously^22^ from HEK293F cells using Nickel charged IMAC Sepharose 6 Fast Flow resin (GE Healthcare), and further purified by size exclusion chromatography. Fractions showing absorption at 280 nm were analyzed by electrophoresis on a 12% SDS-PAGE gel, and stained with colloidal Coomassie. Yields obtained (mg/L culture supernatant volume) were 10.1 for P1243, 3.6 for P1156, 46.4 for P1243:G4S:P1156, and 13.1 for P1156:G4S:1243. N-terminal sequencing of two-warhead proteins was performed using Edman degradation at Alta Bioscience, Birmingham.

### Deglycosylation

Two-warhead CKBPs were prepared as above except that kifunensine was added to a final concentration of 7.5μg/mL following transfection. Purified protein was diluted 1:1 with 40 mM HEPES, 300 mM NaCl and 1 mM TCEP, and then treated with PNGaseF (NEB) and EndoF1 (gift from C. Siebold, STRUBI) at a ratio of 1:1000 and incubated overnight at 37 °C. Degylcosylated protein was purified by size exclusion chromatography. Western blotting was performed using standard protocols^63^, using a mouse monoclonal anti-His primary antibody (R&D Systems) and HRP-tagged goat anti-mouse secondary antibody (Jackson), and Western Lightning ECL Pro (Perkin Elmer kit), and imaged on a BioRad ChemiDoc MP system following the manufacturer’s instructions.

### Biolayer interferometry cross-binding screen

Biolayer interferometry was performed as described previously^22^. Briefly, we used an OctetRed® system using dip and read Ni-NTA biosensors (ForteBio). The assay buffer contained 10 mM Na_2_HPO_4_, 1.8 mM KH_2_PO_4_, 2.7 mM KCl, 500 mM NaCl, 0.1% BSA, 0.002% TWEEN-20, pH 7.4. Assay buffer was used to establish the response baseline (180 seconds). The temperature condition (30 °C) was recommended by the manufacturer. Purified evasin (His-tagged) was immobilized on the Ni-NTA sensor using 1 μM evasin in assay buffer for 500 seconds. The sensor was washed with assay buffer for 60 seconds, and then exposed to 300 nM chemokine in assay buffer to record the association phase. As low affinity binders were identified by this assay, for the two-warheads we used a 100 nM chemokine concentration for the cross-binding screen. The cross-binding screen was performed as a single experiment against all human chemokines with the exception of CCL25, CCL26, and CXCL16, where chemokine non-specifically bound to the sensor, and CXCL17, CXCL4L1, and XCL2, which were not available from Peprotech. The sensor was exposed to assay buffer to measure dissociation. The sensor was regenerated as described previously before re-use^22^. Reference experiments were performed by placing a sensor with evasin alone loaded into buffer to account for baseline drift which was then subtracted. Data was processed in ForteBio Data Analysis 9 software.

### Biolayer interferometry

Titration experiments were carried as described previously^22^. Briefly, each experiment was performed once with chemokine concentration ranging from 300 nM to 0.4 nM, (except for CXCL2 where a range of 600 nM to 1.2 nM was used due to issues with data fitting) and using a non-interacting reference protein (of sequence DGGQRNAICRLPPDEGICRASIPRFYFNPAEGKCSFFIYGGCEGNENNFETIEECEKTCGEPERPSDFEGADFETGCAPKPQRGFCKGFLDHWFFNVTSGECEAFLYSGCGGNDNNYESKEECEIACKLTGGASAWSHPQFEKLEHHHHHHHH) to allow for non-specific binding to the sensor. For each titration assay, at least five chemokine concentrations were studied. ForteBio Data Analysis 9 software was used to process the data and calculate association (k_on_), dissociation (k_off_), and affinity (K_d_) constant using a one-site binding fit. The off-rate (k_off_, s^−1^) obtained from biolayer interferometry was used to calculate the target residence time or dissociation half-life as described^22,64^. For certain chemokine-evasin interactions where a curve fit was not possible, a steady state analysis was performed using ForteBio Data Analysis 9 software to obtain the K_d,_ from the equilibrium response, and here k_off_ and k_on_, and hence target residence times were not calculated. Data with poor curve fits (R^2^ < 0.9) were excluded.

### THP-1 cell migration assays

THP-1 monocyte migration assays were performed as described previously^22^. Briefly, chemokine (EC_80_ dose) and evasin (0.006–300 nM doses, in 0.5% fetal bovine serum) were added to the bottom chamber of a 96-well transwell migration plate (5 μM pore size, Corning) and incubated for 30 min at 37 °C before beginning the cell migration assay by adding 5E5 cells to the top chamber. Three technical replicates for each IC_50_ determination were analysed in GraphPad Prism fitting an inhibitor response curve with 4 parameters. The mean IC_50_ from three biological replicates was then calculated. In control experiments (Fig. S3A) we observed that evasins inhibited the small degree of migration induced in vehicle alone controls, we attribute this to neutralization of naturally occurring chemokines in serum^65^.

### Granulocyte migration assays

Human granulocyte migration assays (see Supplementary Material Figs S3 and S4) were performed using 96 well transwell migration plates (3 μM pore size, Corning). Fresh whole blood was collected from healthy individuals each day of experiment. To collect granulocytes, red blood cells were first depleted, from whole blood, using HetaSep™ (StemCell Technologies) following instructions of the manufacturer. All nucleated cells were then collected and any remaining red blood cells lysed using MACS Red Lysis Buffer (MACS, Miltenyi Biotec). Cells were counted on a ATTUNE flow cytometer using a forward scatter (FSC) versus side scatter (SSC) dot plot. Effective concentrations (EC) EC_80_ and EC_50_ for each chemokine was determined each day, for each batch of blood, in three technical replicates. Chemokines (0–100 nM (Peprotech), in 150 μl RPMI-1640 + L-glu (2 mM) + 0.5% heat treated fetal bovine serum (all from Sigma) were placed in the bottom chamber. Cells (2E5, in 50 μl RPMI- 1640 + L-glu (2 mM) + 0.5% heat treated fetal bovine serum) were placed in the top chamber, and incubated at 37 °C, 5% CO2 for one hour. The migration plate was shaken at 850 r.p.m. for 10 minutes, and media from bottom plate transferred to a U-bottomed 96 well plate. Cells were counted on a ATTUNE flow cytometer using a FSC versus SSC dot plot, and data analysed in GraphPad Prism fitting an agonist response curve with 4 parameters. IC_50_ for each evasin was determined in three technical and three biological replicates using the above system. Chemokine (EC_80_ dose determined on day of experiment) and evasin (0.01 nM-600 nM concentration) were added to the bottom chamber and incubated for 30 min 37 °C, before beginning the cell migration assay. Data (three technical replicates for each IC_50_ determination) were analysed in GraphPad Prism fitting an inhibitor response curve with four parameters. The mean IC_50_ from three biological replicates was then calculated. In control experiments (Supplementary Material Fig. S3B) we observed that evasins inhibited the small degree of migration induced in vehicle alone controls, we attribute this to neutralization of naturally occurring chemokines in serum^65^. The experiments performed on human blood samples were done according to University of Oxford guidelines. Specifically, this study did not require institutional ethical approval as the human tissue was being used solely as a reagent to assay evasin activity. Verbal informed consent was taken from all volunteers who donated the blood samples. All samples were anonymised before use, and no samples were stored. Formal review of the process was obtained from the University of Oxford Medical Sciences Interdivisional Research Ethics Committee (MS IDREC).

### Statistical analyses

GraphPad Prism was used to calculate summary statistics. Tests of significance were calculated using ordinary one-way ANOVA, assuming Gaussian distribution and equal variance, in GraphPad Prism, with Tukey’s correction for multiple testing.

### Data availability

The authors declare that data supporting this manuscript are available either within the manuscript or as Supplementary Information.

### Nucleotide accession numbers

JAG91989.1 (P1243) and JAB72437.1 (P1156)

**Note added in proof**: A further novel C8 evasin that binds CC chemokines was reported by us while this manuscript was under review^66^.

## Electronic supplementary material

Supplementary Information
