# Supplementary material for: Genetically engineered two-warhead evasins provide a method to achieve precision targeting of disease-relevant chemokine subsets
Source: Sci Rep. 2018 Apr 20;8:6333. doi: 10.1038/s41598-018-24568-9 (PMC5910400; doi:10.1038/s41598-018-24568-9)
Supplement: Supplementary file 1 — Supplementary Information [file 41598_2018_24568_MOESM1_ESM.pdf]

Supplementary Material for:

Genetically engineered two-warhead evasins provide a method to achieve precision targeting of disease-relevant chemokine subsets

Yara Alenazi[1], Kamayani Singh[1], Graham Davies[1], James R.O. Eaton[1,2], Philip Elders[1], Akane Kawamura[1,2] & Shoumo Bhattacharya[1*.]

Supplementary Figures:

Figure S1. Homology modelling of P1243.
Figure S2. Cross-binding assays
Figure S3. Effect of CKBPs alone on cell migration.
Figure S4. Human granulocyte migration assay.
Figure S5. Uncropped images of colloidal Coomassie stained SDS-polyacrylamide gels showing purified elutions of P1243, P1156 and two-warhead evasins.
Figure S6. N-terminal sequencing of two-warhead evasins
Figure S7. His western blot of two-warhead evasins
Figure S8. Biolayer interferometry sensorgrams of P1243:G4S:P1156 binding to CXCL4, 7, 12, 13, 14
Table S1. Chemokines expressed in certain diseases that are targeted by the two-warhead evasin P1243:G4S:P1156
Table S2. Summary of data shown in Fig. S2

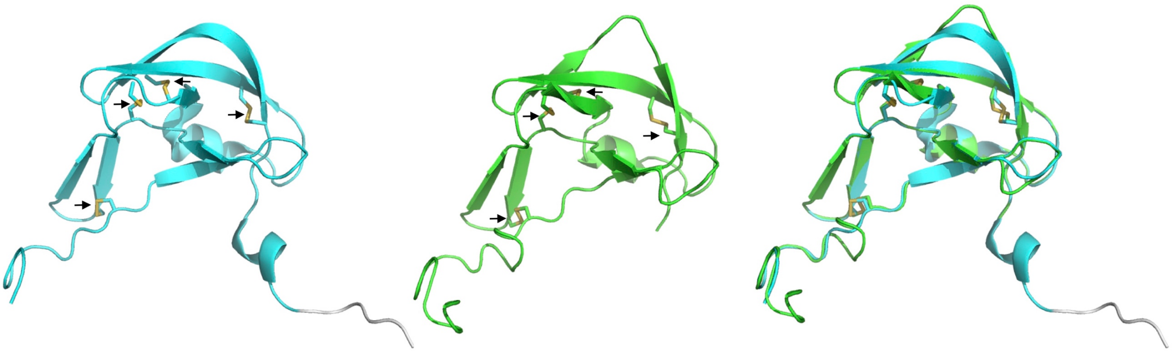

Figure S1. **Homology modelling of P1243.** The left panel shows the 3D structure of EVA1_RHISA, based on 3FPU[1] as a ribbon diagram, with disulfide bonds indicated by arrows. The middle panel shows a model of P1243, created using MODELLER[2], based on the 3FPU template. Disulfide bonds (arrows) were identified using the Protein Interaction Calculator in each model [3]. The right panel is an overlay of EVA1_RHISA structure and the model of P1243.

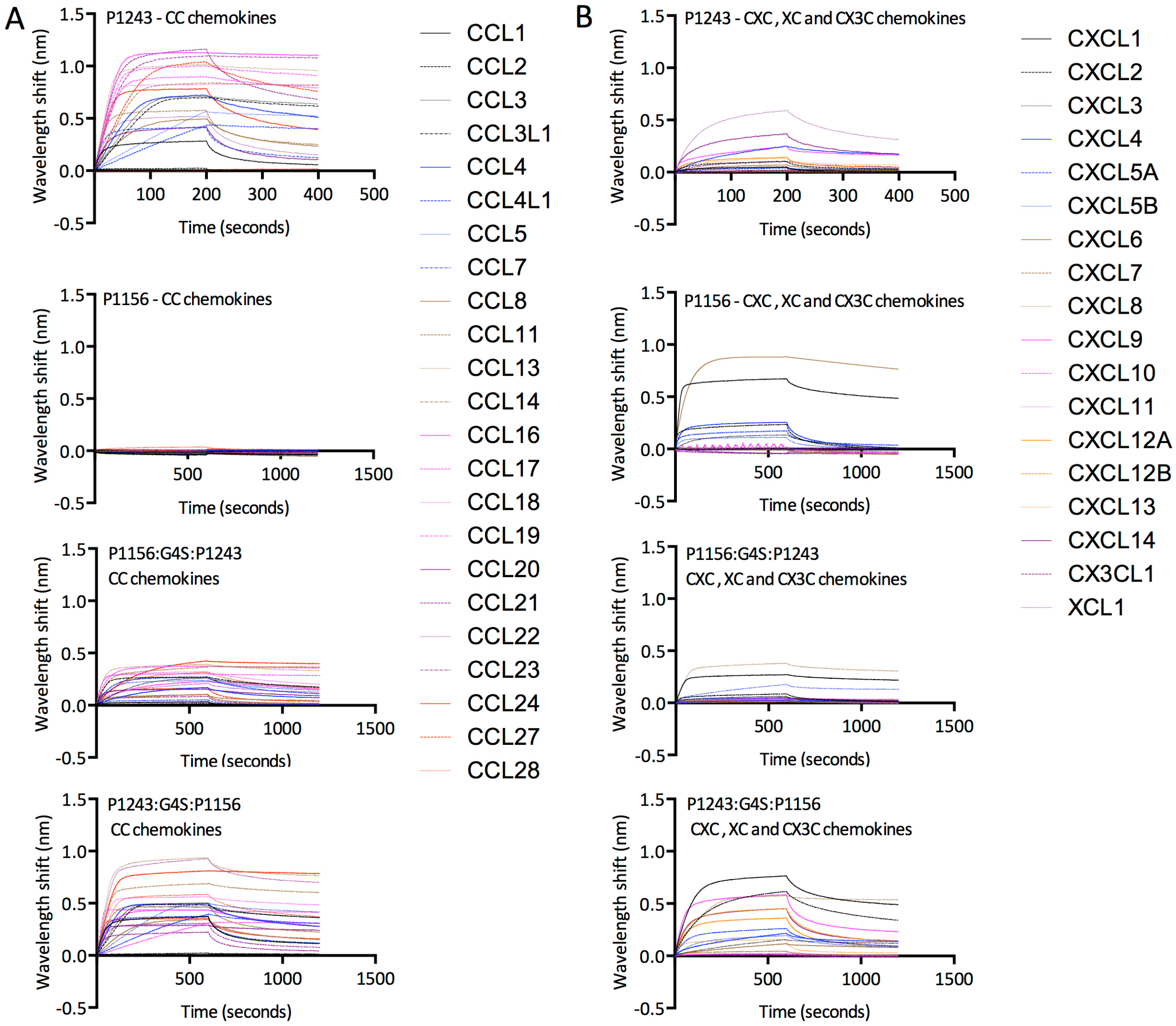

Figure S2. Cross binding assays.

A. Cross binding to CC chemokines for indicated evasins and two warhead constructions. Biolayer interferometry sensorgrams showing binding to different chemokines. Plots display wavelength shift (y-axis, nm) versus time (x-axis, seconds). For P1243 and P1156 a 300 nM chemokine concentration was used, and for the two-warhead evasins a 100nM chemokine concentration was used for screening.

B. Cross binding to CXC, CX3C and XC chemokines for indicated evasins and two warhead constructions. Biolayer interferometry sensorgrams showing binding to different chemokines at 300 nM. Plots display wavelength shift (y-axis, nm) versus time (x-axis, seconds). For P1243 and P1156 a 300 nM chemokine concentration was used, and for the two-warhead evasins a 100nM chemokine concentration was used for screening.

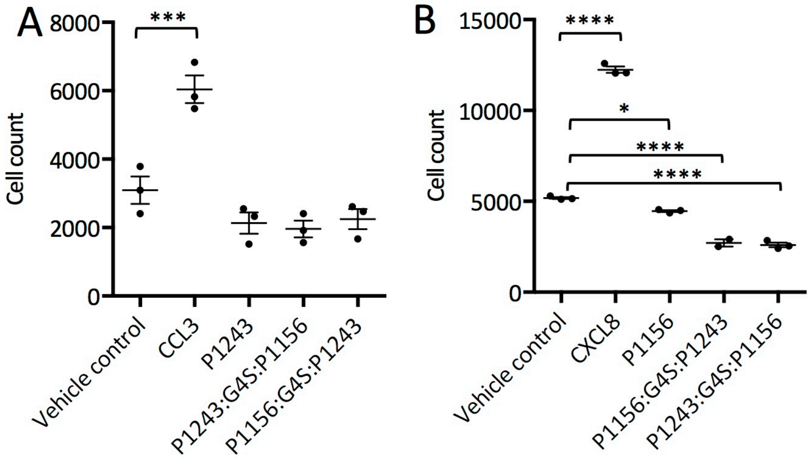

Figure S3. Effect of CKBPs alone on cell migration.
A. Y-axis shows cell count of THP-1 cells migrating through to the bottom chamber in response to vehicle control, CCL3 (EC_{80} dose, positive control), P1243, P1243:G4S:P1156, and P1156:G4S:P1243 (X-axis). Evasins were added at a dose of 100 nM each (the maximum doses used the corresponding IC_{50} experiments). Data are presented as mean ± s.e.m, and individual data points from technical replicates. All samples contained 0.5% fetal bovine serum. A reduction in cell migration was observed in comparison to the vehicle control, but this was not statistically significant.
B. Y-axis shows cell count of granulocyte cells migrating through to the bottom chamber in response to vehicle control, CXCL8 (EC_{80} dose, positive control), P1156, P1243:G4S:P1156, and P1156:G4S:P1243 (X-axis). Evasins were added at doses of 300, 600 and 300 nM respectively (the maximum doses used the corresponding IC_{50} experiments). Data are presented as mean ± s.e.m, and individual data points from technical replicates. All samples contained 0.5% fetal bovine serum. A statistically significant decrease in cell migration was observed for each evasin in comparison to vehicle control.

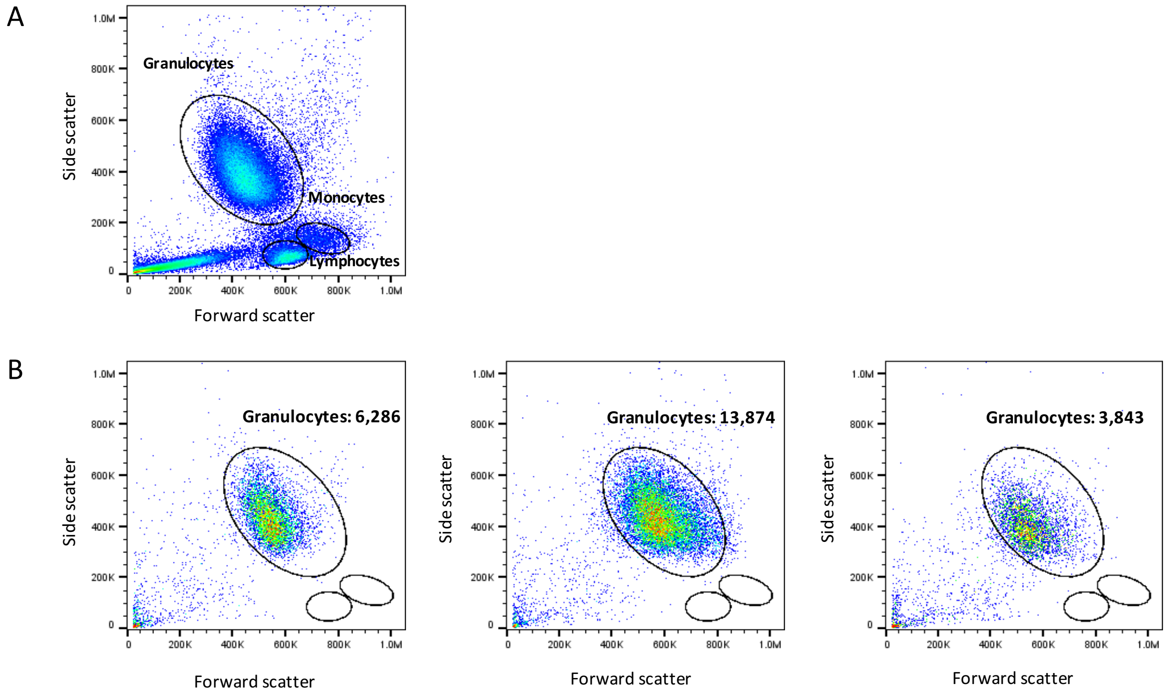

Figure S4. Human granulocyte migration assay.
A. FACS profile characteristics of peripheral blood leukocytes and gating using side scatter (Y-axis) and forward scatter (X-axis). Granulocyte, monocyte and lymphocyte populations are indicated.
B. FACS profile characteristics of peripheral blood leukocyte cell populations that have migrated in response to media alone (left panel), media containing an EC_{80} dose of CXCL8, and media containing an EC_{80} dose of CXCL8 with 100 nM P1156. Granulocyte cell counts are indicated in each panel.

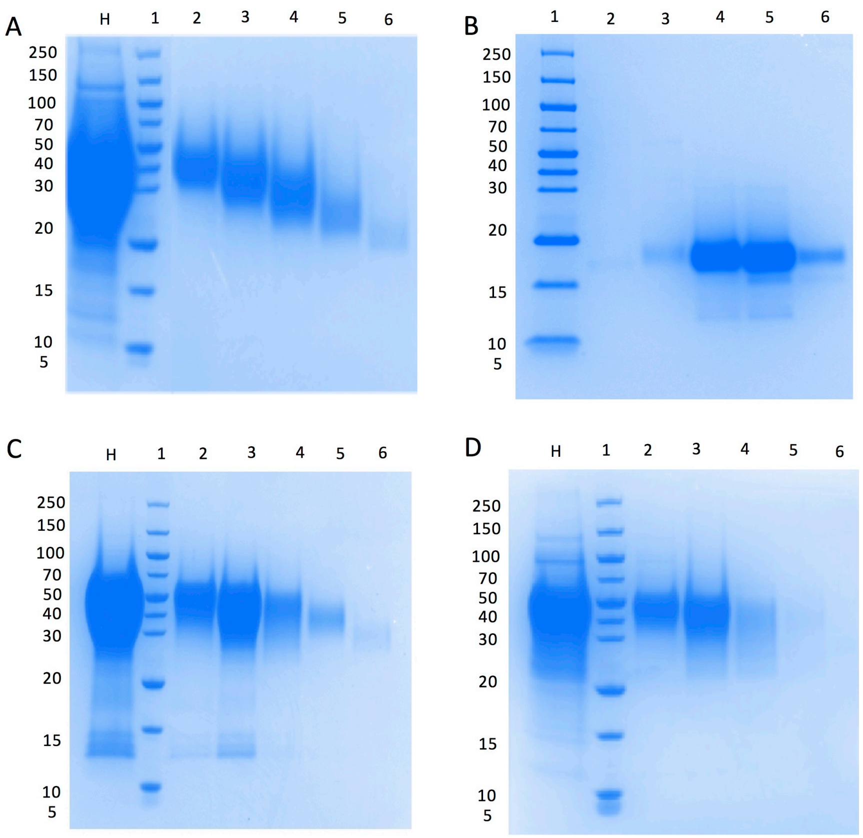

Figure S5. Uncropped images of colloidal Coomassie stained SDS-polyacrylamide gels showing purified elutions of P1243, P1156 and two-warhead evasins.

A. Gel showing size exclusion column fractions (lanes 2-6) obtained following nickel affinity chromatography for P1243. Molecular weight ladder (kDa) is shown in lane 1. Nickel affinity chromatography eluate is shown in lane H.

B. Gel showing size exclusion column fractions (lanes 3-6) obtained following nickel affinity chromatography for P1156. Molecular weight ladder (kDa) is shown in lane 1.

C. Gel showing size exclusion column fractions of P1243:G4S:P1156 obtained following nickel affinity chromatography (lanes 2-6). Molecular weight ladder (kDa) is is shown in lane 1. Nickel affinity chromatography eluate is shown in lane H.

D. Gel showing size exclusion column fractions of P1156:G4S:P1243 obtained following nickel affinity chromatography (lanes 2-6). Molecular weight ladder (kDa) is shown in lane 1. Nickel affinity chromatography eluate is shown in lane H.

P1243:G4S:P1156
ETGRNHTEDNSTEYYDYEEARCACPARHLNNTNGTVLKLLGCHYFCNGTLCTAPDGYPCYNLT
AQQVRTLTTYPNTSCAVGVCMKGTCVKNGTMEQCFKTPGGGGSADDDNELFTVQYCGMNCTK
DEGGTWTGCTGKKEGCKCYHESGKNYGLCLSTEYTDFSQYGNPSDSEIEAAKPKRSDTLSHTGG
GGGSGGGGSGGASAWSHPQFEKLEHHHHHHHH

P1156:G4S:P1243
ETGADDDNELFTVQYCGMNCTKDEGGTWTGCTGKKEGCKCYHESGKNYGLCLSTEYTDFSQY
GNPSDSEIEAAKPKRSDTLSHGGGGSRNHTEDNSTEYYDYEEARCACPARHLNNTNGTVLKLLG
CHYFCNGTLCTAPDGYPCYNLTAQQVRTLTTYPNTSCAVGVCMKGTCVKNGTMEQCFKTPTGG
GGGSGGGGSGGASAWSHPQFEKLEHHHHHHHH

Fig. S6. N-terminal sequencing of two-warhead evasins. Predicted sequences of two warhead evasin proteins reported in this study with N-terminal residues confirmed using Edman degradation shown in red.

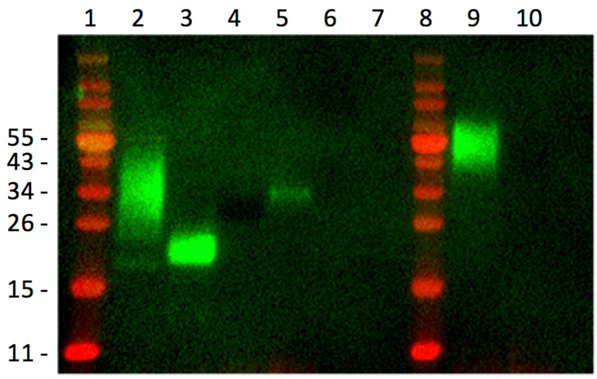

Fig. S7. Western blotting of evasins and two-warheads. Lane 1 and lane 8 show molecular weight markers (kDa). Lane 2 – P1243, lane 3 – P1156, lane 4 – P1243:G4S:P1156 (deglycosylated), lane 5 – P1156:G4S:P1243 (deglycosylated). lane 9 – P1243:G4S:P1156 (glycosylated), lane 10 – P1156:G4S:P1243 (glycosylated). Three ug of indicated proteins were loaded in each lane.

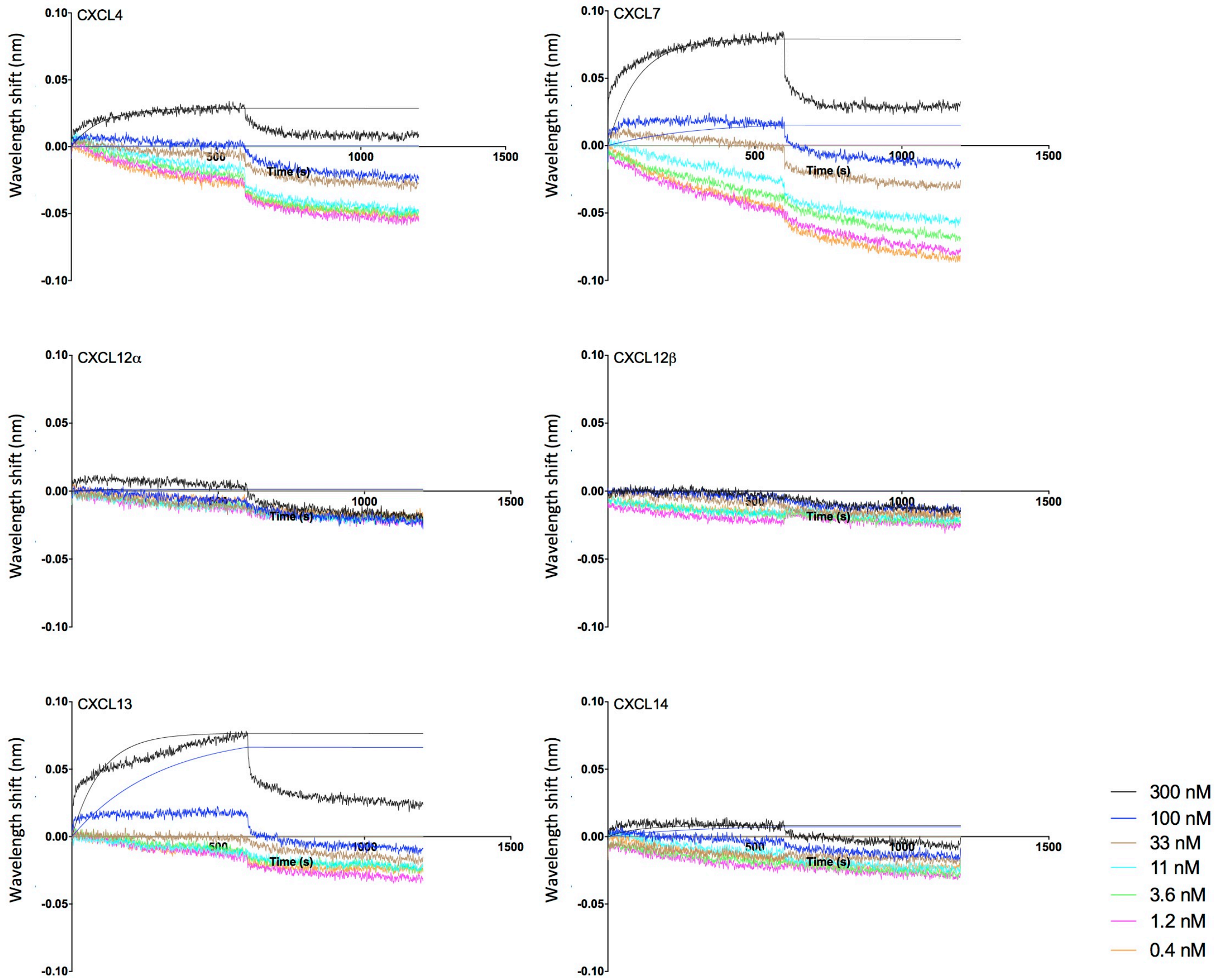

Fig. S8. Biolayer interferometry sensorgrams of P1243:G4S:P1156 binding to indicated chemokines.

Plots display wavelength shift (y-axis, nm) versus time (x-axis, seconds). Solid lines indicate collected data, dashed lines indicate fitted data.

Table S1. Chemokines expressed in certain diseases that are targeted by the two-warhead evasin P1243:G4S:P1156

Disease	Chemokines targeted by P1243:G4S:P1156
Myocarditis	CCL5, CCL13, CCL17, CCL18, CCL19, CXCL8
Myocardial infarction	CCL3, CCL4, CCL5, CCL11, CCL13, CXCL8
Atherosclerosis	CCL3, CCL4, CCL5, CCL11, CCL13, CCL15, CCL17, CCL18, CCL19, CCL23, CXCL8
Vasculitis	CCL3, CCL4, CCL5, CCL13, CCL17, CCL18, CCL19, CXCL1, CXCL8
Idiopathic pulmonary fibrosis	CCL3, CCL4, CCL5, CCL8, CCL11, CCL13, CCL17, CCL18, CCL19, CXCL1, CXCL8
Inflammatory bowel disease	CCL3, CCL4, CCL5, CCL8, CCL11, CCL14, CCL15, CXCL1, CXCL8
Rheumatoid arthritis	CCL3, CCL5, CCL8, CCL13, CCL14, CCL15, CCL17, CCL18, CCL19, CXCL1, CXCL8

The patterns of chemokine expression in each of these diseases is based on literature searches for myocarditis: [4-12]; myocardial infarction: [13, 14], atherosclerosis: [15-29], vasculitis: [30-35], idiopathic pulmonary fibrosis [36-46], inflammatory bowel disease [47-58], rheumatoid arthritis [59].

Table S2. Summary of data shown in Fig. S2

Evasin	Chemokines bound at 100 nM
**P1243**	**CC:** CCL1, CCL3, CCL3L1, CCL4, CCL4L1, CCL5, CCL7, CCL8, CCL1, CCL13, CCL14, CCL15, CCL16, CCL17, CCL18, CCL19, CCL20, CCL21, CCL22, CCL23, CCL24, CCL27<br>**CXC, XC, and CX3C:** XCL1, CX3CL1, CXCL1, CXCL2, CXCL4, CXCL5, CXCL6, CXCL7, CXCL9, CXCL12, CXCL14
**P1156**	**CC:** None<br>**CXC**: CXCL1, CXCL2, CXCL3, CXCL5, CXCL6, CXCL8,
**P1156:G4S:P1243**	**CC:** CCL1, CCL3, CCL3L1, CCL4, CCL4L1, CCL5, CCL7, CCL8, CCL11, CCL13, CCL14, CCL15, CCL16, CCL17, CCL18, CCL19 , CCL20, CCL21, CCL22, CCL23, CCL24, CCL27<br>**CXC:** CXCL1, CXCL2, CXCL3, CXCL5, CXCL8
**P1243:G4S:P1156**	**CC:** CCL1, CCL3, CCL3L1, CCL4, CCL4L1, CCL5, CCL7, CCL8, CCL11, CCL13, CCL14, CCL15, CCL16, CCL17, CCL18, CCL19, CCL20, CCL21, CCL22, CCL23, CCL24, CCL27,<br>**CXC:** CXCL1, CXCL2, CXCL3, CXCL4, CXCL5, CXCL6, CXCL7, CXCL8, CXCL9, CXCL12α, CXCL12β, CXCL13, CXCL14

References

1. Dias, J.M. et al. Structural Basis of Chemokine Sequestration by a Tick Chemokine Binding Protein: The Crystal Structure of the Complex between Evasin-1 and CCL3. *PLoS ONE* **4**, e8514 (2009).
2. Webb, B. & Sali, A. Protein structure modeling with MODELLER. *Methods Mol Biol* **1137**, 1-15 (2014).
3. Tina, K.G., Bhadra, R. & Srinivasan, N. PIC: Protein Interactions Calculator. *Nucleic Acids Res* **35**, W473-476 (2007).
4. Escher, F. et al. Fractalkine in human inflammatory cardiomyopathy. *Heart* **97**, 733-739 (2011).
5. Fuse, K. et al. Enhanced expression and production of monocyte chemoattractant protein-1 in myocarditis. *Clinical and experimental immunology* **124**, 346-352 (2001).
6. Lehmann, M.H., Kuhnert, H., Muller, S. & Sigusch, H.H. Monocyte chemoattractant protein 1 (MCP-1) gene expression in dilated cardiomyopathy. *Cytokine* **10**, 739-746 (1998).
7. Nogueira, L.G. et al. Myocardial chemokine expression and intensity of myocarditis in Chagas cardiomyopathy are controlled by polymorphisms in CXCL9 and CXCL10. *PLoS neglected tropical diseases* **6**, e1867 (2012).
8. Kittleson, M.M. et al. Gene expression in giant cell myocarditis: Altered expression of immune response genes. *International journal of cardiology* **102**, 333-340 (2005).
9. Lassner, D. et al. Improved diagnosis of idiopathic giant cell myocarditis and cardiac sarcoidosis by myocardial gene expression profiling. *Eur Heart J* **35**, 2186-2195 (2014).
10. Satoh, M. et al. Expression of cytokine genes and presence of enteroviral genomic RNA in endomyocardial biopsy tissues of myocarditis and dilated cardiomyopathy. *Virchows Arch* **427**, 503-509 (1996).
11. Zuern, C.S. et al. Endomyocardial expression of SDF-1 predicts mortality in patients with suspected myocarditis. *Clin Res Cardiol* **104**, 1033-1043 (2015).
12. Borst, O. et al. CXCL16 is a novel diagnostic marker and predictor of mortality in inflammatory cardiomyopathy and heart failure. *International journal of cardiology* **176**, 896-903 (2014).
13. de Lemos, J.A. et al. Serial measurement of monocyte chemoattractant protein-1 after acute coronary syndromes: results from the A to Z trial. *Journal of the American College of Cardiology* **50**, 2117-2124 (2007).
14. Orn, S. et al. The chemokine network in relation to infarct size and left ventricular remodeling following acute myocardial infarction. *Am J Cardiol* **104**, 1179-1183 (2009).
15. Haque, N.S. et al. CC chemokine I-309 is the principal monocyte chemoattractant induced by apolipoprotein(a) in human vascular endothelial cells. *Circulation* **102**, 786-792 (2000).
16. Reape, T.J. & Groot, P.H. Chemokines and atherosclerosis. *Atherosclerosis* **147**, 213-225 (1999).
17. Haley, K.J. et al. Overexpression of eotaxin and the CCR3 receptor in human atherosclerosis: using genomic technology to identify a potential novel pathway of vascular inflammation. *Circulation* **102**, 2185-2189 (2000).
18. Yu, R. et al. Involvement of leukotactin-1, a novel CC chemokine, in human atherosclerosis. *Atherosclerosis* **174**, 35-42 (2004).
19. Weber, C. et al. CCL17-expressing dendritic cells drive atherosclerosis by restraining regulatory T cell homeostasis in mice. *J. Clin. Invest.* **121**, 2898-2910 (2011).
20. Calvayrac, O. et al. CCL20 is increased in hypercholesterolemic subjects and is upregulated by LDL in vascular smooth muscle cells: role of NF-κB. *Arteriosclerosis, thrombosis, and vascular biology* **31**, 2733-2741 (2011).
21. Erbel, C. et al. Functional profile of activated dendritic cells in unstable atherosclerotic plaque. *Basic Res Cardiol* **102**, 123-132 (2007).
22. Kimura, S. et al. Expression of macrophage-derived chemokine (CCL22) in atherosclerosis and regulation by histamine via the H2 receptor. *Pathol. Int.* **62**, 675-683 (2012).

23. Kim, C.-S. et al. Potential involvement of CCL23 in atherosclerotic lesion formation/progression by the enhancement of chemotaxis, adhesion molecule expression, and MMP-2 release from monocytes. *Inflamm. Res.* **60**, 889-895 (2011).
24. Abd Alla, J. et al. Angiotensin-converting enzyme inhibition down-regulates the pro-atherogenic chemokine receptor 9 (CCR9)-chemokine ligand 25 (CCL25) axis. *Journal of Biological Chemistry* **285**, 23496-23505 (2010).
25. Zhang, X. et al. Chemokine CX3CL1 and its receptor CX3CR1 are associated with human atherosclerotic lesion volnerability. *Thromb. Res.* **135**, 1147-1153 (2015).
26. Mach, F. et al. Differential expression of three T lymphocyte-activating CXC chemokines by human atheroma-associated cells. *J. Clin. Invest.* **104**, 1041-1050 (1999).
27. Abi-Younes, S. et al. The stromal cell-derived factor-1 chemokine is a potent platelet agonist highly expressed in atherosclerotic plaques. *Circ. Res.* **86**, 131-138 (2000).
28. Smedbakken, L.M. et al. Increased levels of the homeostatic chemokine CXCL13 in human atherosclerosis - Potential role in plaque stabilization. *Atherosclerosis* **224**, 266-273 (2012).
29. Minami, M. et al. Expression of SR-PSOX, a novel cell-surface scavenger receptor for phosphatidylserine and oxidized LDL in human atherosclerotic lesions. *Arteriosclerosis, thrombosis, and vascular biology* **21**, 1796-1800 (2001).
30. Eardley, K.S., Smith, S.W. & Cockwell, P. Chemokines in vasculitis. *Front Biosci (Elite Ed)* **1**, 26-35 (2009).
31. Brix, S.R. et al. CC Chemokine Ligand 18 in ANCA-Associated Crescentic GN. *J Am Soc Nephrol* **26**, 2105-2117 (2015).
32. Dallos, T. et al. CCL17/thymus and activation-related chemokine in Churg-Strauss syndrome. *Arthritis and rheumatism* **62**, 3496-3503 (2010).
33. Eriksson, P., Andersson, C., Cassel, P., Nystrom, S. & Ernerudh, J. Increase in Th17-associated CCL20 and decrease in Th2-associated CCL22 plasma chemokines in active ANCA-associated vasculitis. *Scandinavian journal of rheumatology* **44**, 80-83 (2015).
34. Corbera-Bellalta, M. et al. Blocking interferon γ reduces expression of chemokines CXCL9, CXCL10 and CXCL11 and decreases macrophage infiltration in ex vivo cultured arteries from patients with giant cell arteritis. *Annals of the rheumatic diseases* **75**, 1177-1186 (2016).
35. Blaschke, S., Brandt, P., Wessels, J.T. & Muller, G.A. Expression and function of the C-class chemokine lymphotactin (XCL1) in Wegener's granulomatosis. *J Rheumatol* **36**, 2491-2500 (2009).
36. Hartl, D. et al. A role for MCP-1/CCR2 in interstitial lung disease in children. *Respir Res* **6**, 93 (2005).
37. Schmidt, K. et al. Bronchoalveoloar lavage fluid cytokines and chemokines as markers and predictors for the outcome of interstitial lung disease in systemic sclerosis patients. *Arthritis research & therapy* **11**, R111 (2009).
38. Capelli, A., Di Stefano, A., Gnemmi, I. & Donner, C.F. CCR5 expression and CC chemokine levels in idiopathic pulmonary fibrosis. *Eur Respir J* **25**, 701-707 (2005).
39. Willems, S. et al. Multiplex protein profiling of bronchoalveolar lavage in idiopathic pulmonary fibrosis and hypersensitivity pneumonitis. *Ann Thorac Med* **8**, 38-45 (2013).
40. DePianto, D.J. et al. Heterogeneous gene expression signatures correspond to distinct lung pathologies and biomarkers of disease severity in idiopathic pulmonary fibrosis. *Thorax* **70**, 48-56 (2015).
41. Schupp, J.C. et al. Macrophage activation in acute exacerbation of idiopathic pulmonary fibrosis. *PLoS One* **10**, e0116775 (2015).
42. Petrek, M. et al. The source and role of RANTES in interstitial lung disease. *Eur Respir J* **10**, 1207-1216 (1997).
43. Belperio, J.A. et al. The Role of the Th2 CC Chemokine Ligand CCL17 in Pulmonary Fibrosis. *The Journal of Immunology* **173**, 4692-4698 (2004).

44. Antoniou, K.M. et al. Different angiogenic activity in pulmonary sarcoidosis and idiopathic pulmonary fibrosis. *Chest* **130**, 982-988 (2006).
45. Vasakova, M. et al. Bronchoalveolar lavage fluid cellular characteristics, functional parameters and cytokine and chemokine levels in interstitial lung diseases. *Scand J Immunol* **69**, 268-274 (2009).
46. Vuga, L.J. et al. C-X-C motif chemokine 13 (CXCL13) is a prognostic biomarker of idiopathic pulmonary fibrosis. *Am J Respir Crit Care Med* **189**, 966-974 (2014).
47. Mazzucchelli, L. et al. Differential in situ expression of the genes encoding the chemokines MCP-1 and RANTES in human inflammatory bowel disease. *The Journal of pathology* **178**, 201-206 (1996).
48. Banks, C., Bateman, A., Payne, R., Johnson, P. & Sheron, N. Chemokine expression in IBD. Mucosal chemokine expression is unselectively increased in both ulcerative colitis and Crohn's disease. *The Journal of pathology* **199**, 28-35 (2003).
49. Reinecker, H.C. et al. Monocyte-chemoattractant protein 1 gene expression in intestinal epithelial cells and inflammatory bowel disease mucosa. *Gastroenterology* **108**, 40-50 (1995).
50. Uguccioni, M. et al. Increased expression of IP-10, IL-8, MCP-1, and MCP-3 in ulcerative colitis. *Am J Pathol* **155**, 331-336 (1999).
51. Ahrens, R. et al. Intestinal macrophage/epithelial cell-derived CCL11/eotaxin-1 mediates eosinophil recruitment and function in pediatric ulcerative colitis. *Journal of immunology* **181**, 7390-7399 (2008).
52. Kotarsky, K. et al. A novel role for constitutively expressed epithelial-derived chemokines as antibacterial peptides in the intestinal mucosa. *Mucosal immunology* **3**, 40-48 (2010).
53. Puleston, J. et al. A distinct subset of chemokines dominates the mucosal chemokine response in inflammatory bowel disease. *Alimentary pharmacology & therapeutics* **21**, 109-120 (2005).
54. Papadakis, K.A. et al. CCR9-positive lymphocytes and thymus-expressed chemokine distinguish small bowel from colonic Crohn's disease. *Gastroenterology* **121**, 246-254 (2001).
55. Sans, M. et al. Enhanced recruitment of CX3CR1+ T cells by mucosal endothelial cell-derived fractalkine in inflammatory bowel disease. *Gastroenterology* **132**, 139-153 (2007).
56. Izzo, R.S. et al. Neutrophil-activating peptide (interleukin-8) in colonic mucosa from patients with Crohn's disease. *Scandinavian journal of gastroenterology* **28**, 296-300 (1993).
57. Mahida, Y.R. et al. Enhanced synthesis of neutrophil-activating peptide-1/interleukin-8 in active ulcerative colitis. *Clinical science* **82**, 273-275 (1992).
58. Dotan, I. et al. CXCL12 is a constitutive and inflammatory chemokine in the intestinal immune system. *Inflammatory bowel diseases* **16**, 583-592 (2010).
59. Szekanecz, Z. & Koch, A.E. Successes and failures of chemokine-pathway targeting in rheumatoid arthritis. *Nat Rev Rheumatol* **12**, 5-13 (2016).
